# Exploration of human brain tumour metabolism using pairwise metabolite-metabolite correlation analysis (MMCA) of HR-MAS ^1^H NMR spectra

**DOI:** 10.1371/journal.pone.0185980

**Published:** 2017-10-25

**Authors:** Basetti Madhu, Alexandra Jauhiainen, Sean McGuire, John R. Griffiths

**Affiliations:** 1 Cancer Research UK Cambridge Institute, University of Cambridge, Li Ka Shing Centre, Robinson Way, Cambridge, United Kingdom; 2 Early Clinical Biometrics, AstraZeneca AB R&D, Gothenburg, Sweden; Linköping University, SWEDEN

## Abstract

**Methods:**

We quantified 378 HRMAS ^1^H NMR spectra of human brain tumours (132 glioblastomas, 101 astrocytomas, 75 meningiomas, 37 oligodendrogliomas and 33 metastases) from the eTumour database and looked for metabolic interactions by metabolite-metabolite correlation analysis (MMCA).

**Results:**

All tumour types showed remarkably similar metabolic correlations. Lactate correlated positively with alanine, glutamate with glutamine; creatine + phosphocreatine (tCr) correlated positively with lactate, alanine and choline + phosphocholine + glycerophosphocholine (tCho), and tCho correlated positively with lactate; fatty acids correlated negatively with lactate, glutamate + glutamine (tGlut), tCr and tCho. Oligodendrogliomas had fewer correlations but they still fitted that pattern.

**Conclusions:**

Possible explanations include (i) glycolytic tumour cells (the Warburg effect) generating pyruvate which is converted to lactate, alanine, glutamate and then glutamine; (ii) an association between elevated glycolysis and increased choline turnover in membranes; (iii) an increase in the tCr pool to facilitate phosphocreatine-driven glutamate uptake; (iv) lipid signals come from cytosolic lipid droplets in necrotic or pre-necrotic tumour tissue that has lower concentrations of anabolic and catabolic metabolites. Additional metabolite exchanges with host cells may also be involved. If tumours co-opt a standard set of biochemical mechanisms to grow in the brain, then drugs might be developed to disrupt those mechanisms.

## Introduction

Magnetic Resonance Spectroscopy (MRS) has been widely used for the study and characterisation of brain tumours since it can detect patterns of metabolites that are characteristic of different tumour types. So far, the main interest has been in developing methods for improving the sensitivity and specificity of MRI-based tumour diagnosis by including data from MRS spectra that can be obtained non-invasively during the MRI examination. Two large European Union-funded collaborative research programmes–INTERPRET [1] and eTumour [2]—have built up large and publicly-accessible databases of human brain tumour spectra which can be used for diagnostic purposes. Most of the emphasis in these programmes was on non-invasive diagnosis from *in vivo* ^1^H MRS spectra of the tumours that could be taken during MRI examinations, and the algorithms developed within the INTERPRET programme for classifying those *in vivo* spectra did, indeed, significantly improve radiological diagnosis of several tumour types [3].

Although the non-invasive MRS method used by INTERPRET has obvious advantages for improving diagnosis in a clinical setting, from a scientific standpoint the number of metabolites that can be detected is limited and their concentrations do not provide much insight into the metabolism of the tumours. However, if a sample of the tumour is available (as is usually the case, either from the diagnostic biopsy or from an operation specimen), then much more detailed *ex vivo* ^1^H NMR spectra can be obtained by using the High Resolution Magic Angle Spinning (HRMAS) method, which also has the advantage (compared with solution-state NMR methods which involve chemical extraction of the metabolites) that it is non-destructive, so the sample is available for other studies after the spectrum has been obtained. Such data were obtained by the eTumour project, which followed up and extended the work of INTERPRET. It was funded under EU Framework 6 and ran from 2004–2009, involving 20 collaborating institutions throughout Europe and Argentina (FP6-2002-LIFESCIHEALTH 503094). MRS spectra were accrued from more than a thousand patients including several hundred *ex vivo* HRMAS ^1^H NMR brain tumour spectra that had been obtained from biopsies or operation specimens at 11 of the centres. These spectra are publicly accessible (http://solaria.uab.es/eTumour/) [4] (The eTumour data base http://solaria.uab.es/eTumour/ location has been moved and it is now available through the webpage http://gabrmn.uab.es/), and they provide data from much larger numbers of human tumour samples than are normally available for metabolic studies. We have utilised that eTumour HRMAS ^1^H NMR database for the present study on tumour metabolism.

Metabolite assays by HRMAS ^1^H NMR have the advantage that all the measurements from a specimen are obtained simultaneously with no prior derivatisation; thus one knows the relative concentrations of both aqueous and lipophilic metabolites in that sample with high precision. Such data are ideal for metabolite–metabolite correlation analysis (MMCA) [5–7], a method that allows detection of interactions between the metabolites by exploiting the tiny homeostatic adjustments that maintain the metabolome in a steady state. The concentrations of two metabolites will be correlated positively if samples with a high concentration of the first metabolite also tend to have a high concentration of the second metabolite, while samples with a low concentration for the first metabolite also tend to have low concentrations for the second metabolite. Conversely, if a high concentration of one metabolite in a set of samples is associated with a low concentration of a second metabolite in the same set of samples, then that demonstrates negative correlation between those two metabolites. Because we are concerned only with the covariation of concentrations in a set of samples, MMCA can thus inform on control mechanisms within the metabolome in tissues that were in a steady state, and there is no need to perturb them in any way.

The use of metabolite ratios between related metabolite pairs reduces the overall biological variability in the dataset and thereby increases the statistical power. Systematic experimental errors, such as variance in the concentration of external standards, are cancelled out in ratios, which reduces the overall noise in the dataset. Probably most importantly, when a metabolite pair is connected by a biochemical pathway, metabolite ratios approximate to the corresponding reaction rate under idealized steady state assumptions. Metabolite ratios then represent a biologically most relevant entity, namely the flux through a biochemical pathway [8]. It is possible to visualize the relations between metabolite concentrations in samples by using a scatterplot [7]. Since the metabolites have to maintain homeostasis at all times for routine cellular functions, the plot need not have a fixed metabolite ratio, but the values are rather scattered in the graph. Some of these metabolite pairs can show correlations which are either positive or negative [7]. Using these pairwise metabolite correlations one can also construct metabolic networks[9].

## Material and methods

Ethical approval: All procedures performed in studies involving human participants were in accordance with the ethical standards of the institutional and/or national research committee and with the 1964 Helsinki declaration and its later amendments or comparable ethical standards.

Informed consent: Informed consent was obtained from all individual participants included in the study. The eTumour project time period for recruiting the patients and collecting the patient samples was from 2006 to 2009. Several centres in different countries participated in the research programme. Each centre had to ensure ethical approval by their own local ethics committees under the local regulations.

More details can be found at http://cordis.europa.eu/resul/rcn/50778_en.html

All the HRMAS ^1^H NMR spectral data were downloaded from the eTumour database (http://solaria.uab.es/eTumour/) [4]. The HRMAS ^1^H NMR acquisition protocol in the eTumour project was designed to calibrate the 90-degree pulse for each sample and to use the same value for all acquisition protocols of spectra on that sample. The details of all the brain tumour samples used in this study are summarized in S1 Table. A total of 391 data samples from the eTumour database were used: Water suppressed (acquired using the zgpr pulse sequence in the Bruker software) spectra [total n = 378, glioblastomas (n = 132), astrocytomas (n = 101), meningiomas (n = 75), oligodendrogliomas (n = 37) and metastases (n = 33)], T_2_ filtered CPMG 136 ms spectra [total n = 106, glioblastoma (n = 44), astrocytoma (n = 31), meningioma (n = 19), oligodendrogliomas (n = 7) and metastases (n = 5)] and T_2_ filtered CPMG 30 ms [total n = 391, glioblastomas (n = 143), astrocytomas (n = 100), meningiomas (n = 76), oligodendrogliomas (n = 37) and metastases (n = 35) were downloaded and organized according to the brain tumour types that were represented. The water-suppressed spectra (n = 378) were used for quantification of metabolite concentrations with LCmodel software (version 6.3–0) (http://www.lcmodel.com/lcmodel.shtml), while the T_2_ filtered CPMG spectra (n = 106 at 136 ms and n = 391 at 30 ms) were used for PCA and OPLS-DA studies. All the spectral data analysis was performed using Bruker Topspin 3.2 processing software. The spectra were Fourier transformed, phase corrected (zero and first order) and baseline corrected. Spectral calibration was done using the chemical shift of either the creatine (3.03 ppm) or lactate (1.33ppm) metabolite signals in the spectra. Spectral binning from 0.5 to 4.5 ppm in the chemical shift region with 0.01 ppm intervals was done using the Bruker AMIX software. Binned data were exported from text format to Microsoft Excel spreadsheets. Principle component analysis (PCA) and orthogonal projection of latent structures–discriminant analysis (OPLS-DA) were performed using SIMCA-14 software (Umetrics http://umetrics.com/).

### Quantitative analysis

The following metabolites were quantified in the water-suppressed spectra using a modified LCModel (version 6.3–0) basis set: alanine, choline, creatine, lactate, glutamine, glutamate, glycine, glycerophosphocholine, N-acetylaspartate, phosphocholine, phosphocreatine, taurine, myo-inositol and various lipids/macromolecules [10]. The fitted values for metabolite signals in LCModel output incorporate the content of that basis set and thus can be used for estimation of metabolite concentrations and make it possible to perform MMCA. The metabolite concentrations determined by fitting the spectra in LCModel are not absolute concentrations, but are proportional to the concentrations of the metabolites in the LCModel basis set. To get absolute metabolite concentrations, these LCModel output values would have to be corrected for receiver gain and number of scans and then normalised either to trimethylsilylpropanoic acid (TSP) or unsuppressed water. There was no TSP signal in the water-suppressed spectra, nor was there a corresponding unsuppressed water spectrum in the eTumour database, either of which could have yielded absolute metabolite concentrations. However, to perform MMCA we do not need absolute concentrations, but simply a comparison (scatter plot) of two *relative* metabolite concentrations, similar to a metabolite ratio. The values from LCModel fits are all obtained from the same basis set concentrations and thus the concentration ratios from our LCModel outputs can be compared (Dr Stephen Provencher, personal communication, 20 April 2015). Hence, we used the LCModel output values to create the pairwise metabolite scatter plots.

Cramér-Rao lower bounds (expressed herein as SD%) are widely used as a measure of the reliability of in vivo ^1^H MRS of brain spectra, but there can be problems when they are used as criteria for accepting or rejecting metabolite fittings in LCModel [11, 12]. Although most of metabolite signals in the ex vivo HRMAS ^1^H NMR brain tumour spectra we studied had good signal to noise ratios, some of them included weak signals that resulted in low values for the metabolite peaks and Cramér-Rao lower bounds of more than SD% = 20. Bearing in mind the limitations of Cramér-Rao lower bounds as an index of standard error (LCModel Manual, version 6.3-1L, p132, 11, 12) we have not arbitrarily deleted these lower metabolite values from our data. Instead, we have judiciously compared the signal to noise ratio, metabolite levels, quality of spectrum and precision of the fitting of the peak (as can be seen from the residual) in the LCModel fittings before deciding whether to exclude such data points. In practice, most of the excluded peaks had fitted metabolite levels of either zero or close to zero and had very high Cramér-Rao lower bound values (SD = 999.00%). The absence of a few values in the data does not significantly change the MMCA correlation values as they are obtained by a correlation analysis method that involves robust normalisation [7, 13].

#### Sample weights

We also carried out sample tissue weight normalisation for LC-Model output values and did pair-wise MMCA analysis on those values for comparison. Overall 364 datasets out of total 378 have information about the sample weight. We included this information in detail in S1 Table.

#### Data normalisation for MMCA

The HRMAS ^1^H NMR data have a complex structure, deriving as they do from samples acquired from biopsies or surgical specimens obtained at 11 different centres, which were then used to create HRMAS spectra using ^1^H NMR instruments that operated at 500MHz or 600MHz. We have developed a mixed-model normalisation method in order to overcome the problems of performing MMCA analysis on data types with such complexities[13]. Metabolite levels were log-transformed and metabolites with more than 50% missing values across samples within a tumour type were excluded from the analysis. Samples with more than 80% missing values across metabolites were also removed. A nearest neighbour algorithm was used to impute the remaining missing values (on average ~20% of the data). An extension of our normalization method was then used via fitting a mixed model to the data with metabolites as fixed effects, and random effects for centre as well as separate random sample effects for the two different NMR frequencies used. The normalized data were extracted by using the residuals from the model with the fixed effects added, thus removing variation induced by different centres, samples and frequencies. Pearson correlation coefficients were subsequently estimated for pairs of metabolites using the normalized data. The correlations were filtered initially by correcting the p-values for the Pearson correlations for multiplicity by controlling the false discovery rate using the Benjamini-Hochberg method. A further filtering step was based on bootstrap resampling (of patient samples) in order to check the sensitivity of the metabolite-metabolite correlations to outliers. Correlations that were retained in the p-value filtering step but had a bootstrap confidence interval covering zero were not considered significant. We evaluated the significance of the correlations using the following combinations of p-value cut-off and bootstrap confidence level: 0.001 and 0.999, 0.01 and 0.99, and 0.05 and 0.95.

For two groups of metabolites–the creatine and choline compounds–the NMR peaks could not always be resolved. Consequently, we pooled the data for these two groups to give tCr = phosphocreatine + creatine, and tCho = choline + phosphocholine + glycerophosphocholine. Although we were able to resolve the peaks of glutamate and glutamine in the HRMAS ^1^H NMR spectra, we also tried combining them to give tGlut = glutamate + glutamine, as that had been done in some earlier studies and found that this combination gave biologically interesting results.

### Histopathology of samples

During the eTumour project, after the HRMAS protocol had been completed, samples had been fixed in formalin, stained with haematoxylin and eosin and sent for histopathological review[14]. Tumour type and grade were determined by a histopathologist. The histology was evaluated according to the WHO 2000 classification [4] and the results were recorded in the eTumour database, from which we downloaded them.

## Results

**Fig 1:**
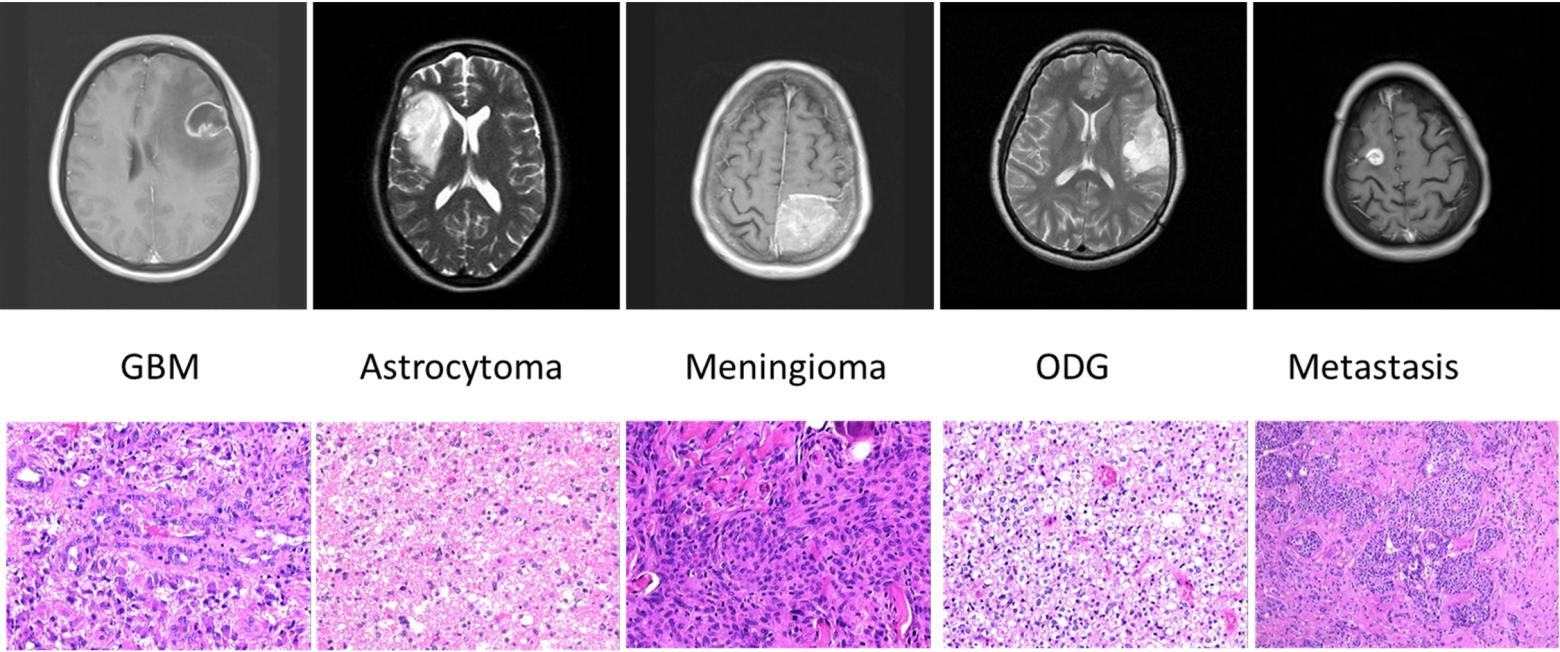
MR images and corresponding H&E sections from five types of brain tumours. The five tumour types illustrated are a glioblastoma (GBM), an astrocytoma (AST), a meningioma (MN), an oligodendroglioma (ODG) and a metastasis (MET) from a mammary carcinoma.

Representative MRI and H&E sections (taken from the eTumour database) from five types of brain tumours are shown in Fig 1. Tumour size and location in the brain can be assessed from the MR images, while H&E sections may show differences in cellularity, depending on the type of tumour.

**Fig 2:**
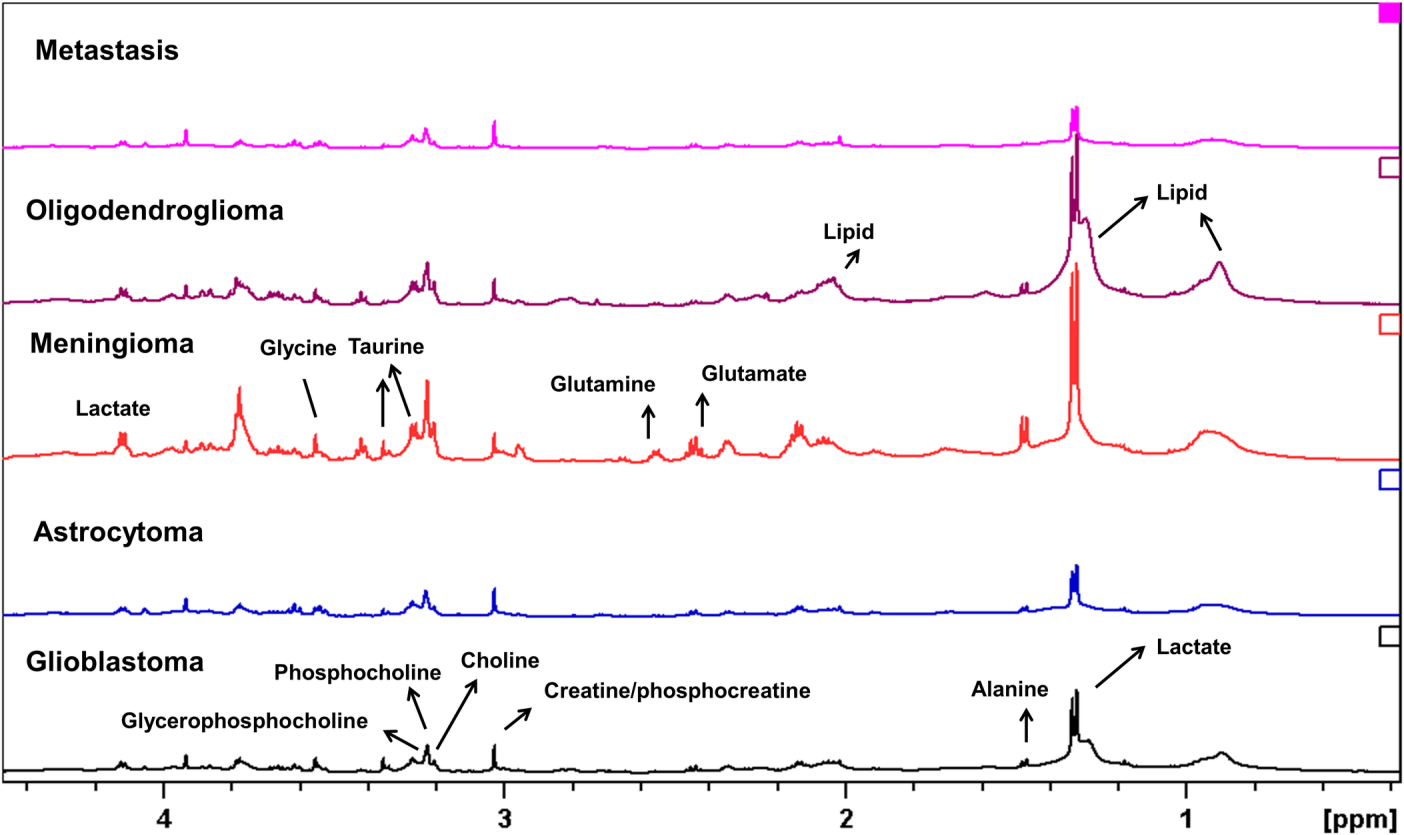
Average HRMAS ^1^H NMR spectra from the five tumour types. From bottom and upwards: glioblastomas (black, n = 132), astrocytomas (blue, n = 101), meningiomas (red, n = 75), oligodendrogliomas (brown, n = 37) and metastases (pink, n = 33).

We first attempted to classify the data using two established methods, PCA and OPLS-DA. Differences in the metabolite profiles of the various tumour types are clearly apparent (Fig 2) but the PCA scores plots (S1 and S2 Figs) of the astrocytoma and meningioma samples showed only a partial separation of the groups (bottom plots of S2 Fig). The scores plot of the astrocytoma and meningioma samples showed better separations in OPLS-DA with the exception of three astrocytoma samples (S3 Fig). The cause of separation of these groups can be seen in the loadings plot in S3 Fig: it is due to the higher metabolite signals of creatine-containing and choline-containing compounds in astrocytomas and higher lactate signals in meningiomas. The correlation and covariance plot (S-plot) from the OPLS-DA analysis of astrocytoma and meningioma samples (S3 Fig) further confirmed that creatine, choline-containing metabolites and lactate as putative biomarkers for separation of the groups. Overall, however, neither PCA nor OPLS-DA was able to provide biochemically meaningful interpretations.

**Fig 3:**
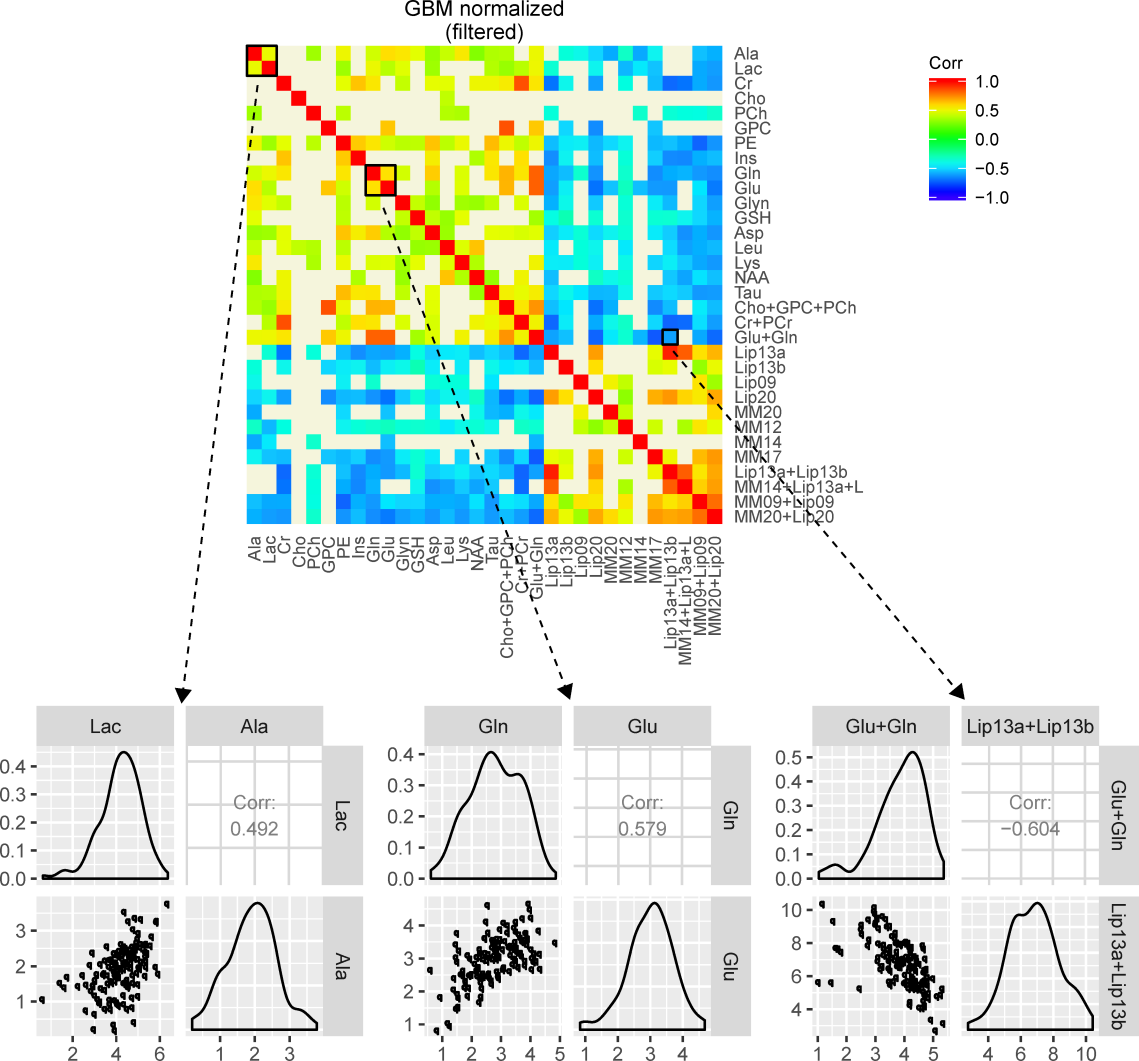
Correlation heatmap and selected metabolite distribution summaries based on glioblastoma datasets. The correlation heatmap shown is normalized and filtered using a p-value cut-off of 0.001 coupled to a bootstrap confidence interval of level 0.999. Metabolites are arranged according to the main biochemical modules. The highlighted squares in the heatmap show the correlations between lactate (Lac) and alanine (Ala), glutamate (Glu) and glutamine (Gln), as well as between tGlut (Glu+Gln) and lipid signals at 1.3ppm (Lip 13a+Lip13b).

**Fig 4:**
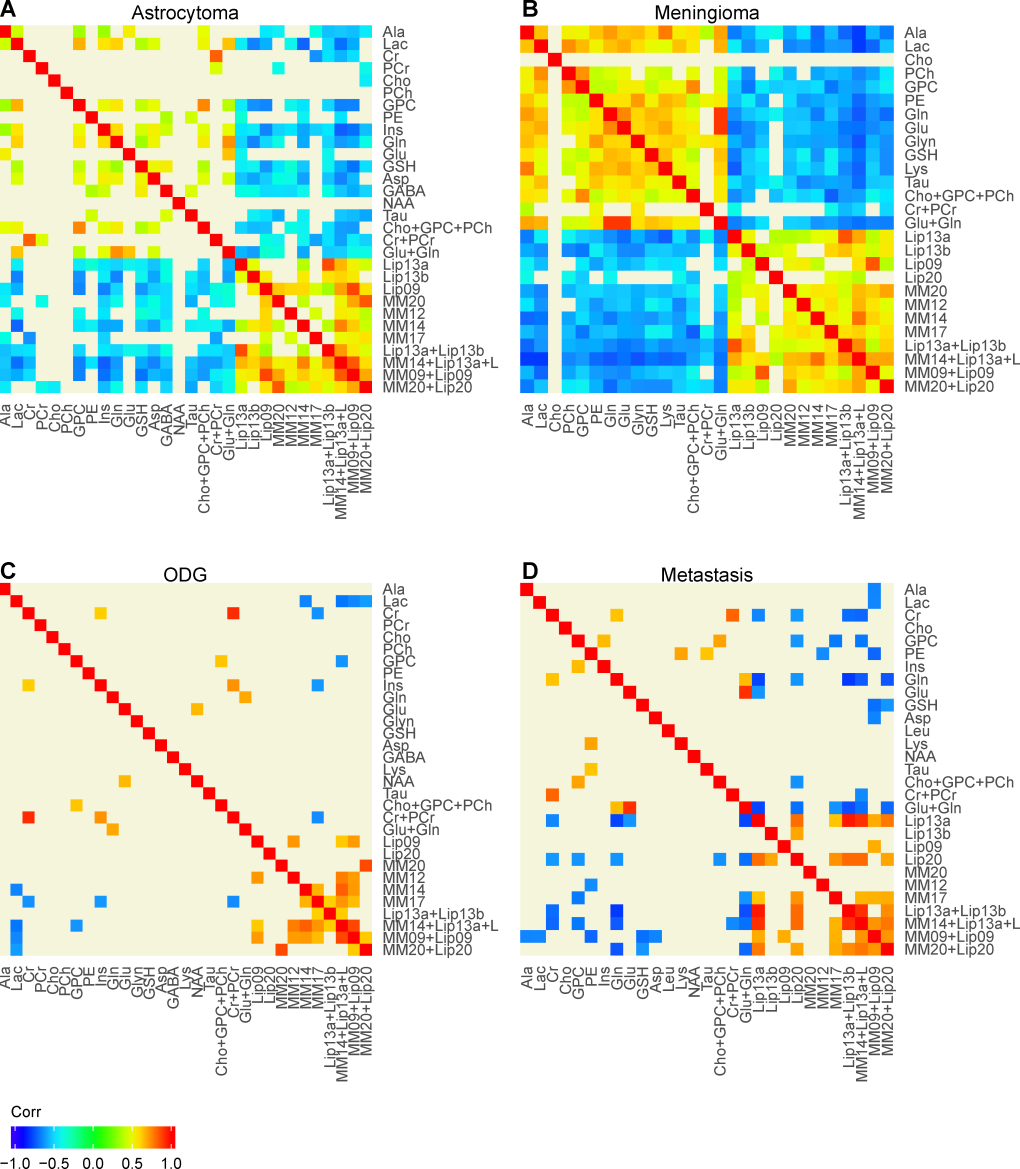
MMCA heatmaps based on datasets from astrocytomas, meningiomas, oligodendrogliomas and metastases. The heatmaps are normalized and filtered using a p-value cut-off of 0.001 coupled to a bootstrap confidence interval of level 0.999. Metabolites are arranged according to the main biochemical modules.

We therefore turned to the NMR-based MMCA method that we have previously described and used to study the metabolism of cultured cells [7]. We estimated the metabolite concentrations in the spectra by using LCModel, and to minimise bias caused by outliers in the data we introduced a bootstrap filter. The highlighted squares in Fig 3 are intended to show the metabolite data (scatter plot, distribution and correlations) forming the basis for the colour coding in the heatmap. For example, the first box in Fig 3, the normalized (filtered) plot of glioblastoma (GBM) data, highlights the metabolite correlations of lactate and alanine, whose details are shown in the first plot in the bottom left. The plot shows the (Gaussian) distribution of the concentrations of lactate and alanine, a scatter plot of lactate versus alanine and the correlation coefficient (in this case 0.492, which is also given in Table 1) between lactate and alanine. This positive correlation of lactate and alanine is represented by a red colour in the heatmap. In a similar way a negative correlation between tGlut and lipids of 1.3ppm appears as a blue colour in the heatmap. This is to emphasize that each box in the heatmap is constructed from the metabolite data of 132 samples and also to show how the correlation coefficient is colour coded in the heatmap. Heatmaps showing pairwise metabolite correlations for the other four types of brain tumours are presented in Fig 4.

**Table 1:** Correlation coefficients of metabolite pairs across the five tumour types.

		GBM	AST	MN	ODG	MET
**Glycolysis**	Lac and Ala	0.492***	0.429***	0.637***	0.199	0.458*
	Lac and (Lip13a+Lip13b)	-0.352**	-0.498***	-0.624***	-0.545**	-0.387*
**Glutamine and glutamate metabolism**	Gln and Glu	0.579***	0.198	0.753***	0.111	0.426*
	tGlut and Lac	0.504***	0.634***	0.667***	0.515**	0.435*
	tGlut and tCho	0.727***	0.384**	0.546***	0.214	0.401*
	tGlut and (Lip1.3a+Lip1.3b)	-0.604***	-0.523***	-0.661***	-0.254	-0.762***
**Energy metabolism**	tCr and Lac	0.39***	0.314*	0.29*	0.452*	0.242
	tCr and Ala	0.27	0.211	0.388***	-0.058	0.323
	tCr and tCho	0.56***	0.336	0.295*	0.272	0.389*
	tCr and (Lip1.3a+Lip1.3b)	-0.719***	-0.485***	-0.555***	-0.58**	-0.591**
**Membrane metabolism**	PCho and GPC	0.023	0.043	0.726***	-0.289	NA
	tCho and Lac	0.327***	0.508***	0.548***	0.208	0.191
	tCho and (Lip1.3a+Lip1.3b)	-0.551***	-0.482***	-0.616***	-0.198	-0.492**
**Lipid metabolism**	Lip1.3a+Lip1.3b and Lip 0.9	0.133	0.343*	0.345**	0.235	0.17
	Lip1.3a+Lip1.3b and Lip 2.0	0.721***	NA	0.449***	-0.249	0.805***

GBM–glioblastoma, AST—astrocytoma, MN—meningioma, ODG- oligodendroglioma, MET—metastasis; Lac–lactate, Ala—alanine, Gln–glutamine, Glu—glutamate, tGlut–glutamine + glutamate, GPC–glycerophosphocholine, PCho–phosphocholine, tCho–choline + phosphocholine + glycerophosphocholine, Lip1.3a and Lip1.3b –fatty acids at 1.3ppm (fatty acids), tCr—creatine + phosphocreatine, Lip 0.9 –fatty acid at 0.9ppm (triglycerides), Lip 2.0 –fatty acid at 2.0ppm. Light blue shade in the row highlights the fatty acids that show negative correlations with metabolites.

The symbol *** represents significance under a multiplicity corrected p-value cut-off of 0.001, with an additional bootstrap filtering step using a confidence level of 0.999, ** represents the same quantity using a cut-off of 0.01 and confidence level of 0.99, and * significance with a cut-off value of 0.05 and confidence level of 0.95. NA–correlation not determined due to low number of values.

For comparison, we also performed the same analysis on data normalized for tissue weight. The properties (median and range) of the sample weights of the five tumour types used in this study (364 datasets out of 378 had weights available) are shown in S4A Fig. Weight-normalised MMCA heatmaps (S4B Fig) were very similar to non-weighted MMCA heatmaps (Figs 3 and 4). We found that the correlations were quite robust with regards to weight normalisation, in the sense that they fluctuated slightly but were not remarkably different to the correlations observed while not compensating for tissue weight. Overall, the p-values were not remarkably different between the two methods (S4C Fig). Hence we present and discuss non-weighted normalised data throughout the study as it contains the data from all 378 datasets (rather than using the reduced number of 364 weight-normalised datasets).

Table 1 shows the correlation coefficients of metabolite pairs in the main metabolic pathways. A correlation coefficient (in absolute terms) (r) of 0.01<r<019 is regarded as a negligible correlation; 0.20<r<0.29 a weak correlation; 0.30<r<0.39 a moderate correlation; 0.40<r<0.69 a good correlation; and r>0.70 an excellent correlation. We have used these terms in the Results section to indicate the strength of each of the correlations. Table 1 also indicates the significance of the correlations with asterisks according to p-value cut-off and bootstrap filtering.

Some positive correlations were observed between metabolites belonging to the same biochemical pathway, but others were found between metabolites in entirely different biochemical modules. In general, the correlations observed in the glioblastomas, astrocytomas, meningiomas and metastases were surprisingly similar. Oligodendrogliomas showed the fewest significant correlations between the metabolites we tested (Table 1 and Fig 4D), but those few correlations still fitted the same basic pattern observed in the other four tumour types.

In the glycolysis-associated metabolites, a good positive correlation between lactate and alanine was observed in all brain tumour types except oligodendrogliomas (S5 Fig and Table 1). In contrast, lactate showed a good to moderate *negative* correlation with the 1.3ppm fatty acid peak in all the 5 tumour types.

The amino acids glutamate and glutamine showed good or excellent positive correlations in glioblastomas, meningiomas and metastases (S6 Fig and Table 1). The combined concentrations of glutamine and glutamate (tGlut) showed a good positive correlation with lactate in all the five tumour types (S7 Fig and Table 1). The tGlut pool also showed good or excellent positive correlations with the tCho pool in glioblastomas, meningiomas and metastases, a moderate correlation in astrocytomas, but only a weak correlation in oligodendrogliomas, whereas it showed good *negative* correlations with the 1.3ppm fatty acid peak in all tumour types except oligodendrogliomas (Table 1).

The tCr pool showed a good correlation with lactate in oligodendrogliomas and moderate positive correlations with lactate in glioblastomas and astrocytomas; a weak but still statistically significant positive correlation was observed between these metabolites in meningiomas. The tCr pool showed a statistically significant moderate correlation with alanine only in meningiomas. It also correlated positively with the tCho pool in glioblastomas, meningiomas (albeit weakly) and metastases but it correlated negatively with the 1.3ppm fatty acid peak in all tumour types (Table 1 and S8 Fig).

Phosphocholine and glycerophosphocholine, two of the membrane-associated choline metabolites, showed an excellent positive correlation, but only in meningiomas. The tCho pool showed a strong positive correlation with lactate in astrocytomas and meningiomas and a moderate but still statistically significant positive correlation in glioblastomas. In contrast, it showed strong *negative* correlations with the 1.3ppm fatty acid peak in glioblastomas, astrocytomas, meningiomas and metastases.

The 1.3ppm lipid signals (hereafter referred to as lipids) showed uniformly negative correlations with the small-molecule metabolites involved in glycolysis (Table 1), membrane metabolism (S9 Fig) energy metabolism (S10 Fig), and glutamine and glutamate metabolism (except in oligodendrogliomas) (Table 1 and S11 Fig).

### Correlations in each tumour type

#### Glioblastomas

**Fig 5:**
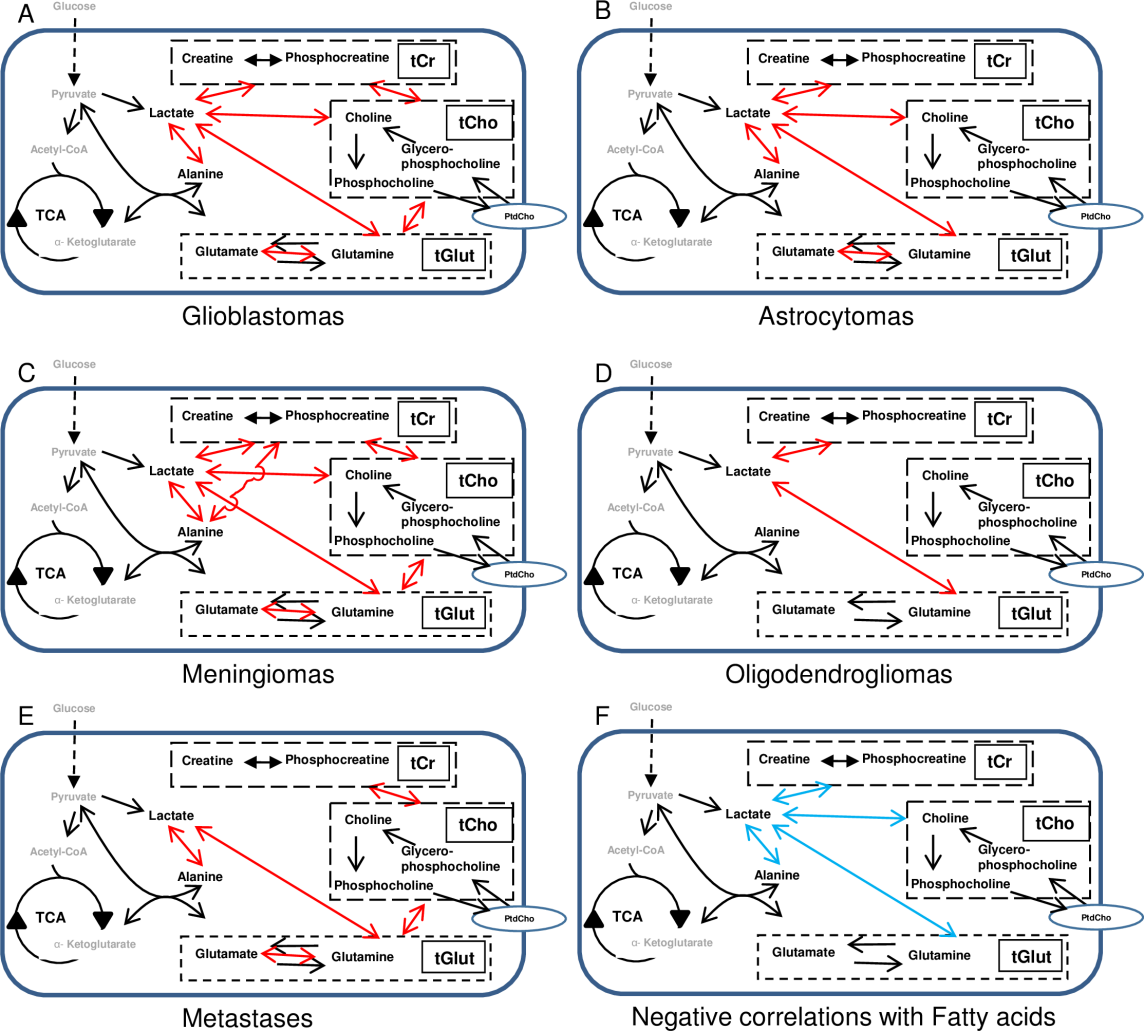
Illustration of the main correlations observed between metabolites across the five tumour types. Positive correlations are shown as red arrows in (A) Glioblastomas; (B) Astrocytomas; (C) Meningiomas; (D) Oligodendrogliomas; and (E) Metastases. (F). Blue arrows indicate the negative correlations between the soluble metabolites and the fatty acid signals that are described in the text. The same pattern was seen in all five tumour types except for oligodendrogliomas, which did not show the correlations between fatty acids and tGlut or tCho (marked as “A” in the diagram). The metabolites detected by HRMAS ^1^H NMR are shown in black with others given in grey. The dashed line boxes indicate the pools of creatine + phosphocreatine (tCr), choline + phosphocholine + glycerophosphocholine (tCho) and glutamate + glutamine (tGlut). PtdCho indicates phosphatidylcholine in cell membranes which is invisible to HRMAS ^1^H NMR.

The statistically significant positive correlations observed in the glioblastoma spectra (Table 1) are diagrammatically illustrated in Fig 5A. The three pools of metabolites, tCr, tCho and tGlut, and their metabolic interconversions, are shown in dashed line boxes. Lactate correlated positively with alanine, tCr, tCho and tGlut. The tCho pool correlated positively with tCr and tGlut, as well as with lactate. Finally, glutamate correlated with glutamine.

#### Astrocytomas

The positive correlations were generally similar in the astrocytoma spectra (Fig 5B): lactate correlated positively with alanine, tCr, tCho and tGlut; tCho correlated positively with tGlut and with lactate. The only major differences from glioblastomas were that glutamate and glutamine were not significantly correlated and nor were tCr and tCho.

#### Meningiomas

Positive correlations in the meningiomas followed the same general pattern (Fig 5C): lactate correlated positively with alanine, tCr, tCho and tGlut, and tCho correlated positively with tCr and tGlut. Like glioblastomas but unlike astrocytomas, correlations were observed between glutamate and glutamine. In contrast to glioblastomas and astrocytomas, tCr correlated positively with alanine and phosphocholine with glycerophosphocholine.

#### Oligodendrogliomas

The exceptions to this general pattern were the oligodendrogliomas (Fig 5D): in those tumours, the only significant positive correlations were between lactate and tGlut and between tCr and lactate. Interestingly, however, these correlations corresponded to the general pattern seen in the other tumour types.

#### Metastases

The positive correlations in the metastases showed a generally similar pattern to those in the glioblastomas, astrocytomas and meningiomas: lactate correlated positively with alanine and tGlut, the tCho pool correlated positively with tCr and tGlut, and glutamate correlated with glutamine.

Overall, the positive biochemical interactions disclosed by the MMCA analysis (see Table 1 and Fig 5) were remarkably similar among four of the five brain tumour types, with oligodendrogliomas showing a reduced number of correlations that still followed the same general pattern.

### Negative correlations

The similarities in all the tumour types between the negative correlations of metabolites with the fatty acids were particularly striking. The statistically significant negative correlations observed in all the 5 tumour types are diagrammatically illustrated in Fig 5F. Fatty acids (actually the 1.3ppm peak due to the–(CH_2_)_n_- groups in fatty acids) correlated negatively with lactate, tCr, tCho and tGlut in all the five tumour types, except that there was no significant correlation between fatty acids and tGlut or tCho in oligodendrogliomas.

## Discussion

NMR data have previously been extensively analysed by pattern recognition methods such as partial least squares regression discriminant analysis (PLS-DA), PCA, and OPLS-DA in order to distinguish normal tissues from malignant tumours and also for classifying different brain tumour types. *In vivo* ^1^H MRS of brain metastases showed that by using the signals from lactate, lipid and choline metabolites the tumours can be categorized into the early, intermediate and late stages of metastases[15]. Creatine has been found to be useful for classifying cultured neuronal and glial cells by pattern recognition analysis of their HRMAS ^1^H NMR spectral profiles [16]. Andronesi et al. attempted to classify brain biopsies by using a HRMAS ^1^H 2D-NMR method called *adiabatic* TOBSY (total correlation through bond spectroscopy) which has provided enhanced sensitivity for several metabolites [17, 18]. Erb et al. used the HRMAS ^1^H NMR metabolomics data from human oligodendroglioma biopsies to classify the grade of malignancy of the tumours [19]. It has also been shown that childhood brain and nervous system tumours can be classified by using the HRMAS ^1^H NMR metabolite profiles obtained from tumour biopsies [20]. By using a bootstrap cross-validation method on *in vivo* ^1^H MRS data of brain tumours from 35 children, medulloblastomas were characterized by high taurine, phosphocholine and glutamate and low glutamine; astrocytomas by low creatine and high N-acetylaspartate; and ependymomas by high myo-inositol and glycerophosphocholine [20]. Cuellar-Baena et al. have used *ex vivo* HRMAS ^1^H NMR metabolite profiles to compare paediatric ependymomas, medulloblastomas and pilocytic astrocytomas and concluded that this information may be useful for assessing tumour grade and determining optimum treatment strategies [21].

Brain tumours are usually heterogeneous, with structures complicated by oedema and necrosis of the adjacent parenchyma; therefore, their spectra are critically affected by voxel size and position. However, in previous studies there was good qualitative agreement between the *in vivo* ^1^H MRS data from human brain tumours obtained by the INTERPRET programme and the data obtained by HRMAS ^1^H NMR analysis of the corresponding brain tumour biopsy [22]. The *ex vivo* human glioblastoma metabolic profiles obtained in the eTumour programme with 1D and 2D NMR methods (COSY, TOCSY and HSQC) comprehensively identified more than 100 resonances, and a good correlation was observed between the *ex vivo* and *in vivo* MRS data [23]. A comparison of the *in vivo* ^1^H MRS metabolite data of brain tumours and *ex vivo* HRMAS ^1^H NMR metabolite data from brain tumour biopsies found a good linear correlation for the majority of metabolites [24]. Opstad et al. also found similar metabolic profiles when comparing *in vivo* ^1^H MRS spectra of brain tumours and the corresponding HRMAS ^1^H NMR profiles obtained from the *ex vivo* tumour biopsies in the eTumour programme [25].

To follow up these promising results we have used the HRMAS ^1^H NMR spectra from 378 human brain tumours samples available in the eTumour database. PCA and OPLS-DA analysis of the spectra (obtained at echo times of both 136 ms and 30 ms) showed no clear classification of the tumours in the scores plot (S1 and S2 Figs). Hence we estimated the metabolite concentrations using LCModel and performed a pairwise metabolite correlation analysis [7] to explore the latent metabolic relations in the samples from these five types of brain tumour.

### Normalization and assessment of significance

We have used an established mixed model method for normalization of the complex metabolite data presented in this paper[7]. Additional components to the mixed model were included to compensate for the different NMR detection frequencies used, making it possible to include metabolites that otherwise would be insufficiently detected at a single frequency. In addition to multiplicity-corrected p-value filtering, a bootstrap filtering step was also employed, since estimated correlation coefficients can be sensitive to outlying observations. By repeatedly sampling the different spectra within each tumour type a large number of times and re-estimating the pairwise correlations for each sample, it is possible to assess the inherent variability of the estimated correlations that is present in the dataset. The bootstrap will, for each correlation, produce an empirical distribution of values. If only a few (outlying) samples contribute to the significance of a certain correlation, this distribution will have a larger spread. The corresponding confidence interval created by taking selected quantiles of the bootstrap distribution will most likely cover zero, indicating that the correlation is not significant. By bootstrap filtering we add a layer of robustness to the assessment of significance. As the different tumour types have different numbers of samples, some tumour types naturally will have more significant correlations than others. When interpreting correlations, it is generally good to keep in mind that although a correlation is significant, it can be of low importance if the estimated coefficient is small.

### Metabolite-metabolite correlation analysis

In our previous studies in which we developed and used an NMR-based MMCA method [7, 12] we attempted to minimise the differences between the samples by obtaining NMR spectra from numerous identical cell cultures, all on the same NMR instrument and using the same pulse sequence. The data for the present paper were much more varied. Spectra were obtained at 11 different centres using 500 MHz and 600 MHz NMR instruments. Furthermore, it is notoriously difficult to standardise the period of warm ischaemia experienced by operation specimens before they are frozen, especially when different surgical teams and experimenters are involved. We therefore investigated how more variable data would affect our normalization procedure and the results from correlation analysis. When the levels of metabolites vary more across samples, simulations indicated that we have a somewhat higher risk of false positives. Due to these observations, we use p-values corrected for multiplicity with an extra bootstrap filtering step added for robustness. The fact that the significant correlations observed have plausible biochemical interpretations and seem to be consistent across most of the tumour types, gives us confidence in the robustness of the MMCA method in this setting.

### Biochemical interpretation

#### Glycolytic metabolism

**Fig 6:**
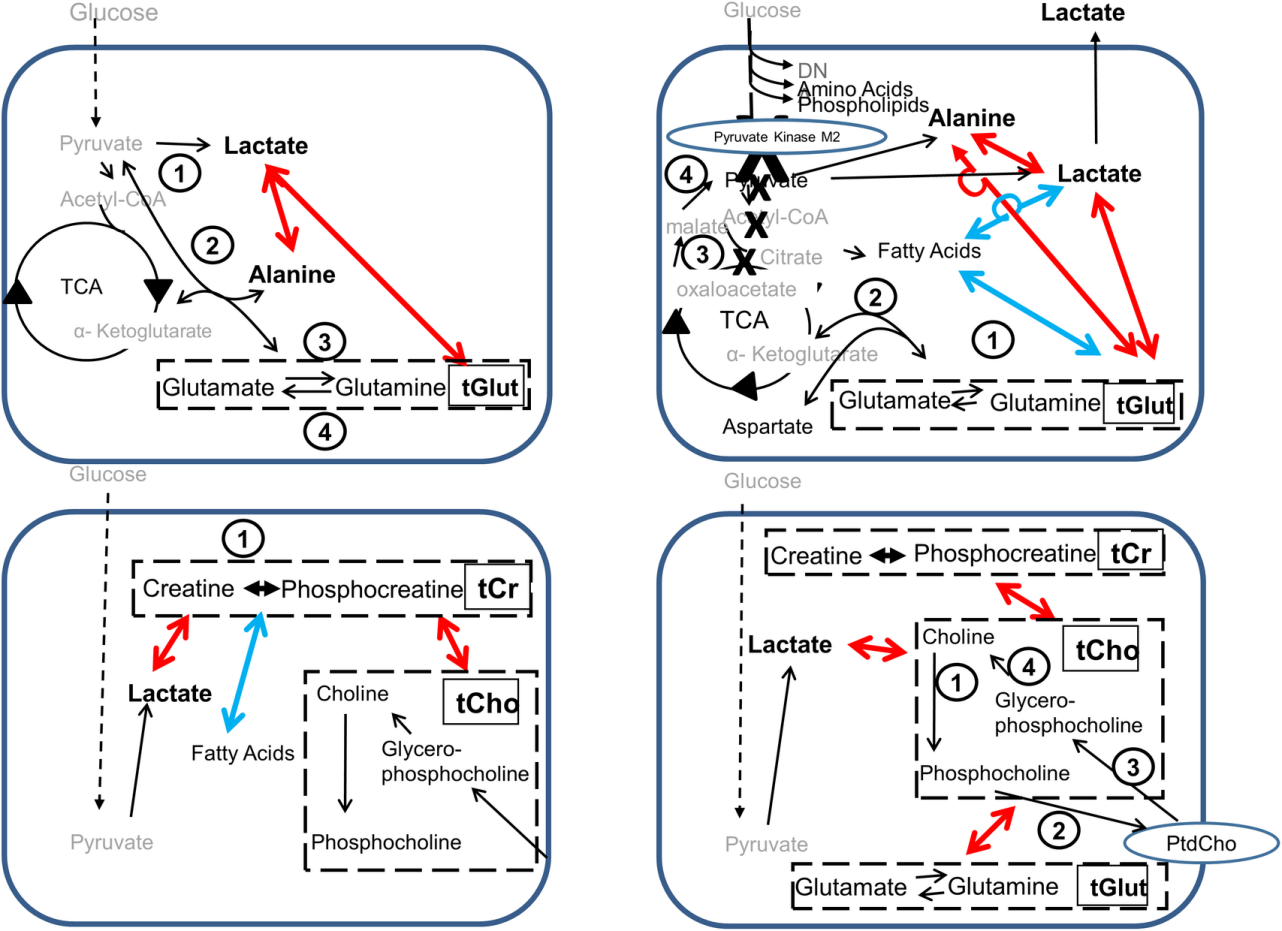
Diagrams illustrating biochemical mechanisms that could give rise to the observed correlations between metabolites. (A) Glycolytic products. The enzymes driving relevant reactions are: 1, lactate dehydrogenase; 2, alanine aminotransferase; 3, glutamine synthetase; 4, glutaminase. (B) Glutaminolysis pathway to bypass PKM2 inhibition. Active enzymes are: 1, glutaminase; 2, aspartate aminotransferase; 3, malate dehydrogenase; 4, malate decarboxylase. (C) Correlations involving the creatine pool. Dashed boxes indicate pools of metabolites: tCr, total creatines (creatine + phosphocreatine); tCho, total cholines (choline + phosphocholine + glycerophosphocholine). The enzyme creatine kinase is marked by the number 1. (D) Correlations involving the choline pool. Dashed boxes indicate pools of metabolites: tCr, total creatines (creatine + phosphocreatine); tCho, total cholines (choline + phosphocholine + glycerophosphocholine); tGlut, glutamate + glutamine pool; PtdCho, phosphatidylcholine. The enzymes marked are: 1, choline kinase; 2, CTP:phosphocholine cytidylyltransferase and choline phosphotransferase; 3, phospholipase Al; phospholipase A2, and lysophospholipase; 4, GPC:choline phosphodiesterase. Metabolites that were not detected by the ^1^H HR-MAS NMR are shown in grey. Positive correlations are shown in red while negative correlations are in blue.

Some of the correlations seem to have intuitively obvious explanations. For instance, in glioblastomas, astrocytomas, meningiomas and metastases there was a positive correlation between lactate and alanine. The positive association between these two metabolites is not surprising since they are two alternative end-products from the glycolytic pathway. A plausible explanation (see Fig 6A) would be that in cells that are producing a large amount of pyruvate from the glycolytic pathway it tends to be converted both to lactate by lactate dehydrogenase

$$\mathrm{Pyruvate}+\mathrm{NADH}\to \mathrm{Lactate}+\mathrm{NA}D^{+}$$ (1)

and to alanine by alanine transaminase

Pyruvate+Glutamate→Alanine+α-Ketoglutarate (2)

Thus both lactate and alanine will tend to be present in high or low concentration, all other things (such as the presence of co-substrates necessary for the synthesis and breakdown reactions) being equal. Increased alanine and lactate levels have previously been observed in brain tumours, and they were also attributed to increased glycolysis [26].

#### Glutamate and glutamine metabolism

Glioblastomas, meningiomas and metastases showed positive correlations between glutamate and glutamine signals. Since glutamine and glutamate can be interconverted by the enzymes glutamine synthetase

$$\mathrm{Glutamate}+\mathrm{ATP}+NH_{3}\to \mathrm{Glutamine}+\mathrm{ADP}+\mathrm{Pi}$$ (3)

and glutaminase,

$$\mathrm{Glutamine}+H_{2}O\to \mathrm{Glutamate}+NH_{3}$$ (4)

it is not surprising that their concentrations seem to rise and fall in unison in some tumour types. The tGlut pool correlated positively with lactate in all tumour types, including oligodendrogliomas, (Fig 5A–5E) whereas in all tumour types there was a negative correlation between tGlut and the 1.3ppm fatty acid peak (Fig 5F). The positive correlation between tGlut and lactate might be connected with the positive correlation between lactate and alanine that was discussed in the previous section. Whenever a molecule of alanine is formed from pyruvate via alanine transaminase a molecule of glutamate will be formed from α-ketoglutarate (Eq 2), and that in turn can be converted (via glutaminase, Eq 4) into glutamine (see Fig 6A). Thus a rise in lactate could be accompanied by a rise in tGlut and *vice versa*.

Rapidly proliferating cancer cells express the PKM2 isoform of the glycolytic enzyme pyruvate kinase. Unlike PKM1, the normal isoform, PKM2 can be inhibited, which blocks pyruvate formation at the end of the glycolytic pathway and conserves glycolytic intermediates for anabolism (see Fig 6B). In these rapidly proliferating cells glutamine can be taken up and converted first to glutamate via glutaminase

$$\mathrm{Glutamine}+H_{2}O\to \mathrm{Glutamate}+NH_{3}$$ (5)

and then to α-ketoglutarate via aspartate aminotransferase

Aspartate+α-Ketoglutarate→Oxaloacetate+Glutamate (6)

followed by a passage through part of the tricarboxylic acid cycle (TCA) to form oxaloacetate which is converted via malate dehydrogenase and malate decarboxylase to pyruvate

$$\mathrm{Malate}+\mathrm{NA}D^{+}\to \mathrm{Oxaloacetate}+\mathrm{NADH}+H^{+}$$ (7)

$$\mathrm{Malate}+\mathrm{NA}D^{+}\to \mathrm{Pyruvate}+CO_{2}+\mathrm{NADH}+H^{+}$$ (8)

which can then be converted to alanine or lactate via Eqs 1 or 2 (see Fig 6B). Once again, the correlation between lactate and alanine can be accounted for, and in this model it is also possible to see why tGlut correlates with both lactate and alanine, since both glutamine and glutamate are involved in the metabolic pathway whereby lactate and alanine can be synthesised. It is also thought that the α-ketoglutarate formed in this pathway can pass backwards (i.e. anticlockwise) through the TCA to citrate which can be bled off to form fatty acids. Both lactate and tGlut are negatively correlated with fatty acids (see Fig 6B). It is easy to see that synthesis of lactate might be inhibited in order to increase flux towards fatty acid formation (and *vice versa*) which would account for this negative correlation. It is not so easy to understand why tGlut, a substrate of both pathways, should be positively correlated with one product, lactate, and negatively correlated with the other, fatty acids. In reality, however, it is thought that solid tumours contain predominantly quiescent cancer cells and that the proliferating fraction is quite small; thus the model illustrated in Fig 6B is likely to represent the behaviour of the main fraction of cancer cells in the tumour and fraction of cells exhibiting the behaviour illustrated in Fig 6B will probably be too small to influence the overall result significantly.

#### Choline metabolism

The choline metabolites are interconverted by a complex network of reactions termed the Kennedy Pathway. Only three of these metabolites—choline, phosphocholine and glycerophosphocholine—could be quantified in the brain tumour spectra, and as their peaks could not always be resolved they are summed as the total choline pool, tCho. The interconversions of these 3 metabolites are illustrated in Fig 6D. Choline forms phosphocholine via choline kinase.

Choline+ATP→Phosphocholine+ADP (9)

Phosphocholine is then converted, via cytidine triphosphate:phosphocholine cytidylyltransferase and choline phosphotransferase, to phosphatidylcholine, which is the major component of biological membranes but is not NMR-visible.

CTP+phosphocholine→⇌pyrophosphate+CDP-choline (10)

CDP-choline+diacylglycerol→phosphatidylcholine+CMP (11)

Phosphatidylcholine can be broken down via phospholipase A2, and lysophospholipase to glycerophosphocholine and then via glycerophosphocholine phosphodiesterase to choline, thus restarting the cycle.

Phosphatidylcholine→Lysophosphatidylcholine+FattyAcid (12)

Lysophosphatidylcholine→Glycerophosphocholine+FattyAcid (13)

Glycerophosphocholine→Choline+Glycerol-3-Phosphate (14)

The NMR-detectable tCho pool correlated positively with lactate, tCr and tGlut, and negatively with fatty acids. These tCho pool correlations imply that the total amount of soluble choline intermediates is biologically significant. A possible explanation for the observed correlation of the size of the tCho pool with the concentrations of the metabolites in these metabolic pathways would be that the tCho pool expands and contracts with variations in the rate of turnover of biological membranes, and that these in turn are correlated with changes in cellular metabolism. The phosphocholine to glycerophosphocholine ratio is regarded as a marker of malignancy[27].

#### Creatine metabolites

The tCr pool displayed some unexpected correlations in several of the tumour types. It was positively correlated with lactate concentration in glioblastomas, astrocytomas and meningiomas (Fig 5A–5C) while in oligodendrogliomas these metabolites showed a moderate correlation that lay just outside statistical significance (p = 0.07). In meningiomas, alanine, another product of glycolysis, was also positively correlated with tCr (Figs 5C and 6C). All five tumour types showed a negative correlation between the tCr pool and the 1.3ppm fatty acid signals (Figs 5F and 6C).

The metabolism of the phosphocreatine-creatine couple has been mainly studied in muscle and to a lesser extent in the brain [28], in which phosphocreatine is thought to provide a reservoir of high energy phosphates that buffers the ATP pool against the sudden energy demands created by muscle contraction or neural transmission. The phosphocreatine-creatine couple is also thought to act as an energy shuttle [29, 30] in which the high energy phosphates of the ATP molecules, created at the inner mitochondrial membrane by oxidative phosphorylation, are transferred (via creatine kinase) to creatine molecules in order to form phosphocreatine. These phosphocreatine molecules then diffuse through the cytosol to places where ATP molecules are being used in energy-requiring reactions and ADP molecules are therefore accumulating. Creatine kinase then transfers the high energy phosphate of phosphocreatine to ADP, forming ATP and allowing the energy-requiring reaction to continue.

Most previous research on phosphocreatine, either in muscle or in the brain, and whether as an energy buffer or as an energy shuttle, has been concerned with its breakdown and resynthesis, and it has been tacitly assumed that the tCr pool stays constant. The present MMCA study challenges that assumption: we have found that the overall size of the tCr pool (i.e. the sum of the concentrations of phosphocreatine and creatine) was positively correlated with lactate concentration in three tumour types (and, alanine, another product of glycolysis, was also positively correlated with tCr in one tumour type), suggesting that there is a hitherto unknown mechanism linking the size of that pool with the concentrations of products of the glycolytic pathway. Furthermore, in all five tumour types there was a negative correlation between the tCr pool and the fatty acid signals.

It is not easy to see why a cancer cell would need phosphocreatine as a reservoir of high energy phosphate, since (in contrast to muscles or neurons) there are no obvious situations in which it would have to upregulate its metabolism within a matter of seconds. It also seems unlikely that the positive correlations we observed with glycolytic products were due to phosphocreatine acting as a shuttle for high energy phosphates, since that would be needed in a cancer cell that was respiring aerobically, which would mean that the glycolytic products would have correlated *negatively* with tCr.

**Fig 7:**
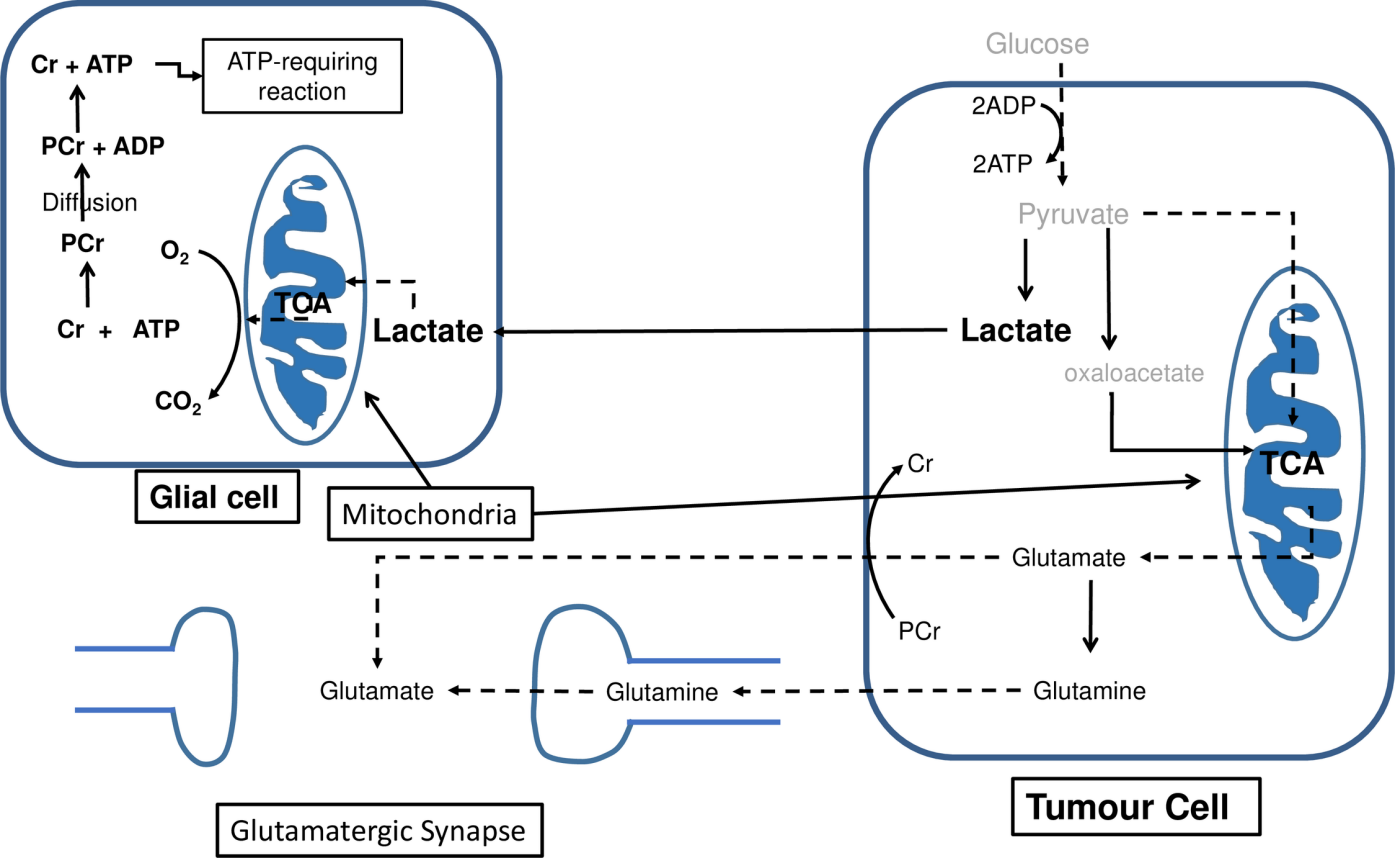
Metabolic interactions between brain tumour cells and host cells. The lactate from tumour cell is transported to glial cells, where it is used a fuel to produce ATP. ATP and PCr are connected via the creatine-kinase reaction. PCr, phosphocreatine; Cr, creatine; TCA, tricarboxylic acid cycle.

One possible explanation for these unexpected correlations would be phosphocreatine-dependent glutamate uptake [31], which is illustrated in Fig 7. Cells that utilise such a mechanism would clearly benefit from matching their tCr pool to their tGlut pool, so a positive correlation would be expected. Another mechanism that might be co-opted by the cancer cells is trafficking of lactate between tumour cells. Cancer cells in solid tumours are in symbiosis with an extracellular milieu that includes T-cells, monocytes, dendritic cells, macrophages, endothelial cells and fibroblasts among many others. Metabolites are exchanged between these cells, so it is theoretically possible that some of the correlations we have observed arise between metabolite pools in different cells. Two metabolite shuttles are of particular importance in brain tumours. The previously-mentioned phosphocreatine-dependent uptake of glutamate forms part of a cyclic glutamate and glutamine shuttle between astrocytes and neurons in the normal brain. It is coupled with the astrocyte-neuron lactate shuttle which supplies its energy demands [32]. Glutamate released at the synapse (neuronal compartment) activates glutamatergic receptors, and it is then taken up by the astrocytes. This will disrupt the sodium ion homeostasis in the astrocytes, which will then be re-established by the action of sodium/potassium ATPase, an energy-consuming process. Glutamate is converted to glutamine in astrocytes by glutamine synthase (Eq 3), which is also an ATP-consuming process, and then it is taken up by neurons where it is converted back to glutamate by glutaminases (Eq 4) [33]. Fig 7 shows a way in which brain tumour cells might utilise these shuttles. If the cancer cells in a tumour co-opt the astrocyte-neuron lactate shuttle to export their glycolytically formed lactate for oxidation in glial cells, as shown in Fig 7, then lactate levels, which would be an aggregate concentration of the lactate pools in the cancer cells, the extracellular space and the glial cells, might correlate positively with the tCr levels in the cytosol of the glial cells. One normally expects cancer cells to be forming lactate for host cells to oxidise, but Lisanti has described a “reverse Warburg effect” in which cancer cells oxidise lactate formed in host cells [34]. In principle, either mechanism could result in a positive correlation between lactate and tCr.

#### Lipid signal correlations

When interpreting the lipid data, it should be remembered that the lipid signals observed in these ^1^H MRS spectra cannot be assigned to distinct species of lipid molecules in the way that is possible for the water-soluble metabolites that we have quantified. Instead, they arise from different types of carbon-hydrogen bond in all fatty acyl chains: 0.9ppm–CH_3_; 1.3ppm–(CH_2_)_n_-; 2.0ppm–CH_2_-CH = CH-CH_2_-. Thus the 1.3ppm signals, for instance, could represent the aggregate of the–(CH_2_)_n_- groups in acyl chains of numerous different types of lipid molecule. The positive correlations observed between the 0.9ppm signals and the 1.3ppm signals, and between the 1.3ppm and 2.0ppm signals probably indicate structural characteristics of fatty acid chains–e.g. that lipid molecules with–CH_3_ and–CH_2_-CH = CH-CH_2_- groups also tend to have–(CH_2_)_n_- groups.

NMR-detectable lipid signals are frequently observed in all types of brain tumours both *in vivo* and *ex vivo*, and lipid droplets are also frequently found by histology, particularly in glioblastomas. A strong correlation between the lipid signal at 1.3ppm and the number of lipid droplets has been observed with Nile-red strained H&E sections in human gliomas of different grades [35]. There was also a positive correlation between increasing necrosis in the H&E sections and the 1.3ppm fatty acid signal; larger lipid droplets were found in frankly necrotic tissue but smaller ones were present in pre-necrotic tissue and the authors conclude that droplets are initially formed in viable hypoxic tissue before necrosis and that they remain within necrotic tissue following cell death [35]. In a rodent brain tumour model undergoing gene therapy, increased water diffusion was observed by ^1^H MRS and also accumulation of NMR visible mobile lipids due to cell death [36]. MR-detectable intra-tumoural lipids, which are mobile lipids that accumulate in high-grade brain tumours, particularly in the necrotic regions, have been proposed as a potential biomarker for the assessment of treatment response in cancer [37].

An unsupervised pattern recognition method used on HR-MAS spectra from human brain metastases showed clustering due to the primary origin of the metastases, mainly based on differences in the fatty acid signals at 1.3 and 0.9 ppm [38]. When these data were subjected to supervised PLS-DA, spectra of the patients who had died less than 5 months after surgery appeared to cluster together in the scores plot [38]. A positive correlation has been shown between fatty acid signals at 1.3ppm and the number of lipid pseudo-droplets (per mm^2^) in human brain tumour tissues classified as no-necrosis, low necrosis and high necrosis [35]. Cheng et al. have found that amount of lipids observed at 0.90ppm in HRMAS ^1^H NMR spectra of glioblastoma specimens showed a positive correlation with necrosis [39]. A significant correlation between lipid signals and necrosis in brain metastases was observed (p < 0.01), irrespective of their primary origin. Increased polyunsaturated fatty acids (constituents of cell and mitochondrial membranes) have been observed in gliomas during apoptosis [26]. Metastatic melanomas showed higher amounts of glycerophosphocholine than other brain metastases [40].

A very consistent finding in the MMCA plots was the negative correlations of the 1.3ppm fatty acid signals with lactate, tGlut, tCr and tCho (see Table 1 and Fig 4); this was observed in four of the five tumour types, while oligodendrogliomas had significant negative correlations of the fatty acids with lactate and tCr but not with tGlut or tCho. A possible explanation for these almost uniform negative correlations is that the fatty acids that give rise to quantifiable HR-MAS ^1^H NMR signals arise mainly from the lipid droplets that form in necrotic tissue or pre-necrotic brain tumour tissue [27] (fatty acid components of biological membranes are held rigidly and thus give rise to broad signals) and that tumour samples with high concentrations of lipid droplets have less viable tissue containing lactate, tGlut, tCr and tCho, and *vice versa*. While that sounds like a plausible explanation for the negative correlations between fatty acids and tGlut, tCr and tCho, the negative correlation with lactate is harder to understand since there are many reports in the literature that lactate is found along with lipids in necrotic and pre-necrotic regions of brain tumours (most of these studies have been on gliomas) both *in vivo* [41] (e.g. by MRS) and *ex vivo* [39]. Those papers would lead one to expect a *positive* correlation between lipid and lactate signals, whereas we observed a *negative* correlation. We considered the possibility that the lactate and lipid signals had been inadequately resolved in the HRMAS ^1^H NMR spectra that we quantified (as can happen with *in vivo* MRS spectra), but that does not appear to be the case, so we have no explanation for this apparently contradictory result.

In general, lipids and/or lipid droplets have frequently been observed in NMR studies on tumours and cancer cells undergoing stress, hypoxia, necrosis, apoptosis, and anti-cancer treatment [26, 37]. The hypothesis that we have proposed to explain the consistently negative correlations between metabolite groups and lipids that we have observed in the five types of brain tumours—that cells with strong metabolic pathway flux (and thus high concentrations of lactate, tCr, tGlut and tCho) tend not to contain NMR-visible lipid droplets, and *vice versa*—suggests a possible mechanism for explaining the phenomena observed in earlier studies.

### Is the MMCA pattern in brain tumours common to tumours in general?

**Fig 8:**
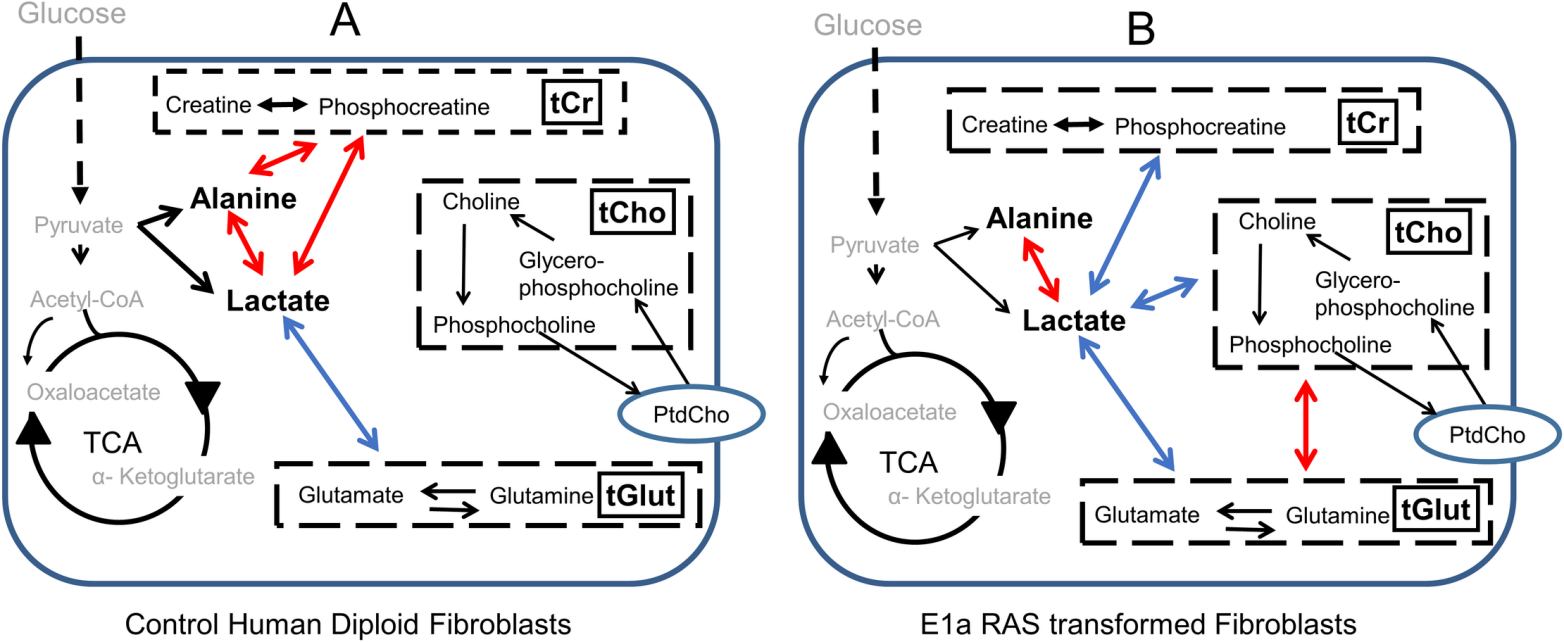
Metabolite-Metabolite correlations in cultured human diploid fibroblasts. A). Control cells B). E1A-Ras transformed cultured cells. Data adapted from Madhu et al **[7]**.

This question cannot currently be answered, since to the best of our knowledge the MMCA method has not been applied to any other type of tumour. Our previous MMCA study was concerned with the biochemical changes we observed in cultured human diploid fibroblasts when they were transformed by the oncogene E1A-Ras [7]; such a transformation is a major step on the way to creation of tumour cells. The cultured cells were chemically extracted, which resulted in the loss of the lipid components but enabled us to detect more soluble metabolites than is possible by HRMAS ^1^H NMR; for instance, we could distinguish the individual components of the tCr and tCho pools. In order to see whether the resulting MMCA data showed a pattern of correlations similar to that seen in the brain tumour spectra we have combined the data from the creatines, cholines and glutamate + glutamine so as to produce tCr, tCho and tGlut pools, and we have calculated their correlations with the other soluble metabolites that were detected in the HRMAS ^1^H NMR spectra, both for the control human diploid fibroblasts and for the E1A-Ras transformed cells (data presented in S3 Table). The results are shown in Fig 8A and 8B; note that the fatty acids were not detected so are not included. In the control cells (Fig 8A), lactate showed positive correlations with alanine and tCr, and alanine also correlated positively with tCr. However, lactate was *negatively* rather than positively correlated with the tGlut pool. In the E1A-Ras transformed cells we saw a very different pattern to that in the brain tumours (Fig 8B). Lactate still correlated positively with alanine, but it correlated *negatively* rather than positively with tCr; furthermore, lactate correlated negatively with tCho and tGlut, and tGlut correlated positively with tCho (Fig 8B and S3 Table). The positive correlation observed between tGlut and tCho was remarkably similar in both the E1A-Ras transformed cells and all the brain tumour types except oligodendrogliomas (Table 1, S3 Table, Figs 5 and 8B), indicating a strong coupling between glutaminolysis and membrane metabolism of cells on the way to transformation and also in established malignancy. In contrast, the negative correlations of lactate with tCho and tGlut observed in E1A-Ras transformed cells were found to be positive when they were present in the brain tumours. In the control human diploid fibroblasts, we observed positive interactions between tCr and both lactate and alanine, similar to our results in several of the brain tumour types. However, in the E1A-Ras transformed cells lactate correlated *negatively* rather than positively with tCr. Since our previous experiment was performed in cell culture we were able to measure both glucose uptake and lactate output, and both increased after E1A-Ras transformation. So in that model, in contrast to the positive correlation observed in the brain tumours, there was a *negative* correlation of lactate with tCr when the cultured cells were transformed and developed a Warburg effect.

E1A-Ras transformed cells do not display all the characteristics of cancer cells, and our study was focussed on the immediate biochemical changes induced by transformation rather than on long-term effects. Nevertheless, as far as they go, the results in Fig 8B do not suggest that the constant biochemical pattern we have observed in the brain tumours is a general characteristic of cancer cells.

## Summary

We have found that 378 HRMAS ^1^H NMR spectra from five types of human brain tumour, display a common pattern of metabolic correlations. Tumours with high levels of lactate tended to have high levels of alanine, tCr, tGlut and tCho; conversely, tumours with high levels of NMR-detectable fatty acids tended to have *low* levels of lactate, tCr, tGlut and tCho. Finally, the creatine pool (tCr) correlated positively with lactate in three tumour types and negatively with NMR-detectable fatty acids in all five tumour types.

We suggest that these correlations are due to the utilisation of a common set of biochemical mechanisms by these brain tumours: (i) Tumour cells that are obtaining energy by glycolysis convert glucose to pyruvate which can be further converted to lactate and alanine; the latter process also forms glutamate which can be converted to glutamine. These effects also tend to be accompanied by high concentrations of NMR-detectable choline compounds (tCho) which could be due (ii) to an association between elevated anaerobic glycolysis and an increase in biological membrane turnover. All these Warburg effect-associated mechanisms are *negatively* correlated with high concentrations of HRMAS ^1^H NMR-detectable fatty acids. We suggest that this could be because (iii) cells that are synthesising fatty acids tend not to rely on glycolytic energy production and vice versa. Further sets of unexpected correlations were found between the creatine pool (tCr) and both the glycolytic products (positive) and the fatty acids (negative). The positive correlations between tCr and glycolytic products could be due to (iv) the cancer cells utilising phosphocreatine-dependent glutamate uptake and/or (v) to the cancer cells exporting lactate to oxidative host cells that utilise the creatine shuttle. Finally, (vi) we suggest that cells that are forming NMR-visible lipids do not utilise phosphocreatine to take up glutamate and *vice versa*.

Remarkably, the general outlines of this complex biochemical pattern were observed in all the types of brain tumour investigated: glioblastomas, astrocytomas, meningiomas and oligodendrogliomas, and in metastases from several types of primary tumour. The pattern was less obvious in the oligodendrogliomas but some of it was still evident. The similarity in biochemistry between glioblastomas and astrocytomas is not entirely unexpected since they are generally thought to arise from the same type of cell, the astrocyte; indeed, some astrocytomas develop into glioblastomas. Meningiomas, in contrast, are considered to develop from cells in the membranes surrounding the brain, and oligodendrogliomas from cells that give rise to the insulating sheaths of axons, so it is harder to see why they should all display similar biochemical patterns. One possible reason for the reduced number of significant correlations in the oligodendroglioma data is the small sample size: there were only 37 oligodendroglioma spectra compared with 135 from glioblastomas, 101 from astrocytomas and 75 from meningiomas. The metastases, however, displayed the common biochemical pattern more prominently than oligodendrogliomas, although their sample size (n = 33) was even smaller; furthermore, the metastases were derived from several different types of primary tumour (see S1 Table). In this context it is interesting to note that there is also a remarkable consistency between the MRS spectra obtained *in vivo* from glioblastomas and MRS spectra obtained from cerebral metastases due to all types of primary tumour. Indeed it is so difficult to distinguish MRS spectra of glioblastomas from those of metastases that special methods have been devised [42].

One possible explanation for the results reported in the present paper would be that all these disparate brain tumour types are co-opting the same set of biochemical mechanisms–some perhaps involving interactions with host cells—in order to grow in the specialised environment of the brain. A possible alternative interpretation comes from recent suggestions that the neural stem cell, astrocyte, and oligodendrocyte precursor cell can all serve as the cell of origin for glioblastomas [43]. If it were to be found that other brain tumour types can also arise from the same set of cell types, then that might explain why they all have such similar biochemistry. However, it would not explain our observation of similar correlations in cerebral metastases that arose from many different types of primary tumour elsewhere in the body, which suggests that utilisation by all brain tumours of a common set of survival mechanisms is the more likely explanation.

If the uniform set of biochemical mechanisms that we have found in many types of human brain tumour is necessary for their growth, then it might be possible to develop novel classes of anticancer drugs in order to disrupt those mechanisms.

## Supporting information

S1 Table Details of the brain tumour datasets used in this study. Numbers of samples with information on tissue weights are given in the brackets.
(PDF)

S2 Table Brain tumour survival rates. Data from http://www.cancer.org/index and www.abta.org/.
(PDF)

S3 Table Correlation coefficients of metabolites in control and E1A/RAS treated samples. Data adapted from ref [7].
(PDF)

S1 Fig PCA scores and loadings plots from the data of HRMAS ^1^H NMR profiles (CPMG with 136 ms). 1. Glioblastoma (green dots, n = 44), 2. Astrocytoma (violet dots, n = 31), 3. Meningioma (red dots, n = 19), 4. Oligodendroglioma (yellow dots, n = 7) and 5. Metastasis (blue dots, n = 5) samples.
(TIF)

S2 Fig PCA scores and loadings plots from the data of HRMAS ^1^H NMR profiles (CPMG with 30 ms). 1. Glioblastomas (green dots, n = 145), 2. Astrocytoma (violet dots, n = 101), 3. Meningioma (red dots, n = 75), 4. Oligodendroglioma (yellow dots, n = 37) and 5. Metastasis (blue dots, n = 33).
(TIF)

S3 Fig OPLS-DA of HRMAS ^1^H NMR profiles (CPMG with 30 ms) data. Astrocytoma (violet dots, n = 101), Meningioma (red dots, n = 75) samples.
(TIF)

S4 Fig Summary of analysis on tissue weighted data. (A) Boxplots illustrating the median and range for tissue weights across the five different tumour types. (B) MMCA heatmaps based on the tissue normalised data from the five tumour types. (C) Comparison of non-weighted and weighted correlation coefficients and p-values across the five tumour types. The heatmaps were filtered using a p-value cut-off of 0.001 coupled to a bootstrap confidence interval of level 0.999.
(PDF)

S5 Fig Correlations observed between glycolytic pathway products (Lac and Ala) across the five different tumour types. (TIF)

S6 Fig Correlations observed between glutamine (Gln) and glutamate (Glu) across the five different tumour types. (TIF)

S7 Fig Correlations observed between tGlut (glutamate + glutamine) content and lactate across the five different tumour types. (TIF)

S8 Fig Correlations between energy metabolism (tCr) and membrane metabolism (tCho) across the five different tumour types. (TIF)

S9 Fig Correlations between membrane metabolites (tCho) and lipids at 1.30 ppm across the five different tumour types. (TIF)

S10 Fig Correlation between energy metabolites (tCr) and lipids at 1.30 ppm across the five different tumour types. (TIF)

S11 Fig Correlation of tGlut (glutamate + glutamine) content and lipid signals at 1.30ppm across the five different tumour types. (TIF)

## References

[1] Julia-Sape M, Griffiths JR, Tate RA, Howe FA, Acosta D, Postma G, et al Classification of brain tumours from MR spectra: the INTERPRET collaboration and its outcomes. NMR Biomed. 2015;28(12):1772–87. doi: 10.1002/nbm.3439 . 26768492 10.1002/nbm.3439

[2] Garcia-Gomez JM, Luts J, Julia-Sape M, Krooshof P, Tortajada S, Robledo JV, et al Multiproject-multicenter evaluation of automatic brain tumor classification by magnetic resonance spectroscopy. MAGMA. 2009;22(1):5–18. doi: 10.1007/s10334-008-0146-y ; PubMed Central PMCID: PMCPMC2797843. 18989714 10.1007/s10334-008-0146-y PMC2797843

[3] Julià-Sapé M, Arias-Mendoza, Fernando, Griffiths, John R. Clinical Trials of MRS Methods. eMagRes: John Wiley & Sons, Ltd; 2015.

[4] Julia-Sape M, Lurgi M, Mier M, Estanyol F, Rafael X, Candiota AP, et al Strategies for annotation and curation of translational databases: the eTUMOUR project. Database (Oxford). 2012;2012:bas035 doi: 10.1093/database/bas035 ; PubMed Central PMCID: PMC3504476. 23180768 10.1093/database/bas035 PMC3504476

[5] Steuer R, Kurths J, Fiehn O, Weckwerth W. Interpreting correlations in metabolomic networks. Biochem Soc Trans. 2003;31(Pt 6):1476–8. 10.1042/. doi: 10.1042/ . 14641093 10.1042/bst0311476

[6] Steuer R, Kurths J, Fiehn O, Weckwerth W. Observing and interpreting correlations in metabolomic networks. Bioinformatics. 2003;19(8):1019–26. . 12761066 10.1093/bioinformatics/btg120

[7] Madhu B, Narita M, Jauhiainen A, Menon S, Stubbs M, Tavare S, et al Metabolomic changes during cellular transformation monitored by metabolite-metabolite correlation analysis and correlated with gene expression. Metabolomics. 2015;11(6):1848–63. doi: 10.1007/s11306-015-0838-z ; PubMed Central PMCID: PMCPMC4605990. 26491426 10.1007/s11306-015-0838-z PMC4605990

[8] Petersen AK, Krumsiek J, Wagele B, Theis FJ, Wichmann HE, Gieger C, et al On the hypothesis-free testing of metabolite ratios in genome-wide and metabolome-wide association studies. BMC Bioinformatics. 2012;13:120 doi: 10.1186/1471-2105-13-120 ; PubMed Central PMCID: PMCPMC3537592. 22672667 10.1186/1471-2105-13-120 PMC3537592

[9] Armitage EG, Kotze HL, Williams KJ. Network-Based Correlation Analysis of Metabolic Fingerprinting Data. Correlation-based network analysis of cancer metabolism SpringerBriefs in Systems Biology: Springer New York; 2014 p. 21–34.

[10] Piccirillo SG, Dietz S, Madhu B, Griffiths J, Price SJ, Collins VP, et al Fluorescence-guided surgical sampling of glioblastoma identifies phenotypically distinct tumour-initiating cell populations in the tumour mass and margin. Br J Cancer. 2012;107(3):462–8. doi: 10.1038/bjc.2012.271 ; PubMed Central PMCID: PMCPMC3405212. 22722315 10.1038/bjc.2012.271 PMC3405212

[11] Tisell A, Leinhard OD, Warntjes JB, Lundberg P. Procedure for quantitative (1)H magnetic resonance spectroscopy and tissue characterization of human brain tissue based on the use of quantitative magnetic resonance imaging. Magn Reson Med. 2013;70(4):905–15. doi: 10.1002/mrm.24554 . 23169203 10.1002/mrm.24554

[12] Bolliger CS, Boesch C, Kreis R. On the use of Cramer-Rao minimum variance bounds for the design of magnetic resonance spectroscopy experiments. Neuroimage. 2013;83:1031–40. doi: 10.1016/j.neuroimage.2013.07.062 . 23933043 10.1016/j.neuroimage.2013.07.062

[13] Jauhiainen A, Madhu B, Narita M, Narita M, Griffiths J, Tavare S. Normalization of metabolomics data with applications to correlation maps. Bioinformatics. 2014;30(15):2155–61. doi: 10.1093/bioinformatics/btu175 . 24711654 10.1093/bioinformatics/btu175

[14] Wright AJ, Fellows GA, Griffiths JR, Wilson M, Bell BA, Howe FA. Ex-vivo HRMAS of adult brain tumours: metabolite quantification and assignment of tumour biomarkers. Mol Cancer. 2010;9:66 doi: 10.1186/1476-4598-9-66 ; PubMed Central PMCID: PMC2858738. 20331867 10.1186/1476-4598-9-66 PMC2858738

[15] Sijens PE, Levendag PC, Vecht CJ, van Dijk P, Oudkerk M. 1H MR spectroscopy detection of lipids and lactate in metastatic brain tumors. NMR Biomed. 1996;9(2):65–71. doi: 10.1002/(SICI)1099-1492(199604)9:2<65::AID-NBM397>3.0.CO;2-N . 8887370 10.1002/(SICI)1099-1492(199604)9:2<65::AID-NBM397>3.0.CO;2-N

[16] Griffin JL, Bollard M, Nicholson JK, Bhakoo K. Spectral profiles of cultured neuronal and glial cells derived from HRMAS (1)H NMR spectroscopy. NMR Biomed. 2002;15(6):375–84. doi: 10.1002/nbm.792 . 12357551 10.1002/nbm.792

[17] Andronesi OC, Mintzopoulos D, Struppe J, Black PM, Tzika AA. Solid-state NMR adiabatic TOBSY sequences provide enhanced sensitivity for multidimensional high-resolution magic-angle-spinning 1H MR spectroscopy. Journal of Magnetic Resonance. 2008;193(2):251–8. doi: 10.1016/j.jmr.2008.05.017 18556227 10.1016/j.jmr.2008.05.017

[18] Andronesi OC, Blekas KD, Mintzopoulos D, Astrakas L, Black PM, Tzika AA. Molecular classification of brain tumor biopsies using solid-state magic angle spinning proton magnetic resonance spectroscopy and robust classifiers. Int J Oncol. 2008;33(5):1017–25. doi: 10.3892/ijo_00000000 ; PubMed Central PMCID: PMCPMC2658602. 18949365 10.3892/ijo_00000000 PMC2658602

[19] Erb G, Elbayed K, Piotto M, Raya J, Neuville A, Mohr M, et al Toward improved grading of malignancy in oligodendrogliomas using metabolomics. Magn Reson Med. 2008;59(5):959–65. doi: 10.1002/mrm.21486 . 18429037 10.1002/mrm.21486

[20] Wilson M, Davies NP, Brundler MA, McConville C, Grundy RG, Peet AC. High resolution magic angle spinning 1H NMR of childhood brain and nervous system tumours. Mol Cancer. 2009;8:6 doi: 10.1186/1476-4598-8-6 ; PubMed Central PMCID: PMC2651110. 19208232 10.1186/1476-4598-8-6 PMC2651110

[21] Cuellar-Baena S, Morales JM, Martinetto H, Calvar J, Sevlever G, Castellano G, et al Comparative metabolic profiling of paediatric ependymoma, medulloblastoma and pilocytic astrocytoma. Int J Mol Med. 2010;26(6):941–8. . 21042791 10.3892/ijmm_00000546

[22] Barton SJ, Howe FA, Tomlins AM, Cudlip SA, Nicholson JK, Bell BA, et al Comparison of in vivo 1H MRS of human brain tumours with 1H HR-MAS spectroscopy of intact biopsy samples in vitro. MAGMA. 1999;8(2):121–8. . 10456375 10.1007/BF02590529

[23] Martinez-Bisbal MC, Marti-Bonmati L, Piquer J, Revert A, Ferrer P, Llacer JL, et al 1H and 13C HR-MAS spectroscopy of intact biopsy samples ex vivo and in vivo 1H MRS study of human high grade gliomas. NMR Biomed. 2004;17(4):191–205. doi: 10.1002/nbm.888 . 15229932 10.1002/nbm.888

[24] Wilson M, Davies NP, Grundy RG, Peet AC. A quantitative comparison of metabolite signals as detected by in vivo MRS with ex vivo 1H HR-MAS for childhood brain tumours. NMR Biomed. 2009;22(2):213–9. doi: 10.1002/nbm.1306 . 19067434 10.1002/nbm.1306

[25] Opstad KS, Wright AJ, Bell BA, Griffiths JR, Howe FA. Correlations between in vivo (1)H MRS and ex vivo (1)H HRMAS metabolite measurements in adult human gliomas. J Magn Reson Imaging. 2010;31(2):289–97. doi: 10.1002/jmri.22039 . 20099340 10.1002/jmri.22039

[26] Griffin JL, Kauppinen RA. A metabolomics perspective of human brain tumours. FEBS J. 2007;274(5):1132–9. doi: 10.1111/j.1742-4658.2007.05676.x . 17298437 10.1111/j.1742-4658.2007.05676.x

[27] Glunde K, Bhujwalla ZM, Ronen SM. Choline metabolism in malignant transformation. Nat Rev Cancer. 2011;11(12):835–48. doi: 10.1038/nrc3162 ; PubMed Central PMCID: PMCPMC4337883. 22089420 10.1038/nrc3162 PMC4337883

[28] Andres RH, Ducray AD, Schlattner U, Wallimann T, Widmer HR. Functions and effects of creatine in the central nervous system. Brain Res Bull. 2008;76(4):329–43. doi: 10.1016/j.brainresbull.2008.02.035 . 18502307 10.1016/j.brainresbull.2008.02.035

[29] Bessman SP, Geiger PJ. Transport of energy in muscle: the phosphorylcreatine shuttle. Science. 1981;211(4481):448–52. . 6450446 10.1126/science.6450446

[30] Gabr RE, El-Sharkawy AM, Schar M, Weiss RG, Bottomley PA. High-energy phosphate transfer in human muscle: diffusion of phosphocreatine. Am J Physiol Cell Physiol. 2011;301(1):C234–41. doi: 10.1152/ajpcell.00500.2010 ; PubMed Central PMCID: PMCPMC3129826. 21368292 10.1152/ajpcell.00500.2010 PMC3129826

[31] Xu CJ, Klunk WE, Kanfer JN, Xiong Q, Miller G, Pettegrew JW. Phosphocreatine-dependent glutamate uptake by synaptic vesicles. A comparison with atp-dependent glutamate uptake. J Biol Chem. 1996;271(23):13435–40. . 8662761 10.1074/jbc.271.23.13435

[32] Belanger M, Allaman I, Magistretti PJ. Brain energy metabolism: focus on astrocyte-neuron metabolic cooperation. Cell Metab. 2011;14(6):724–38. doi: 10.1016/j.cmet.2011.08.016 . 22152301 10.1016/j.cmet.2011.08.016

[33] Magistretti PJ, Allaman I. A cellular perspective on brain energy metabolism and functional imaging. Neuron. 2015;86(4):883–901. doi: 10.1016/j.neuron.2015.03.035 . 25996133 10.1016/j.neuron.2015.03.035

[34] Pavlides S, Whitaker-Menezes D, Castello-Cros R, Flomenberg N, Witkiewicz AK, Frank PG, et al The reverse Warburg effect: aerobic glycolysis in cancer associated fibroblasts and the tumor stroma. Cell Cycle. 2009;8(23):3984–4001. doi: 10.4161/cc.8.23.10238 . 19923890 10.4161/cc.8.23.10238

[35] Opstad KS, Bell BA, Griffiths JR, Howe FA. An investigation of human brain tumour lipids by high-resolution magic angle spinning 1H MRS and histological analysis. NMR Biomed. 2008;21(7):677–85. doi: 10.1002/nbm.1239 . 18186027 10.1002/nbm.1239

[36] Liimatainen T, Hakumäki JM, Kauppinen RA, Ala-Korpela M. Monitoring of gliomasin vivoby diffusion MRI and1H MRS during gene therapy-induced apoptosis: interrelationships between water diffusion and mobile lipids. NMR in Biomedicine. 2009;22(3):272–9. doi: 10.1002/nbm.1320 19009568 10.1002/nbm.1320

[37] Delikatny EJ, Chawla S, Leung DJ, Poptani H. MR-visible lipids and the tumor microenvironment. NMR Biomed. 2011;24(6):592–611. doi: 10.1002/nbm.1661 ; PubMed Central PMCID: PMC3640643. 21538631 10.1002/nbm.1661 PMC3640643

[38] Sjobakk TE, Johansen R, Bathen TF, Sonnewald U, Juul R, Torp SH, et al Characterization of brain metastases using high-resolution magic angle spinning MRS. NMR Biomed. 2008;21(2):175–85. doi: 10.1002/nbm.1180 . 17542042 10.1002/nbm.1180

[39] Cheng LL, Anthony DC, Comite AR, Black PM, Tzika AA, Gonzalez RG. Quantification of microheterogeneity in glioblastoma multiforme with ex vivo high-resolution magic-angle spinning (HRMAS) proton magnetic resonance spectroscopy. Neuro Oncol. 2000;2(2):87–95. ; PubMed Central PMCID: PMC1919517. 11303625 10.1093/neuonc/2.2.87 PMC1919517

[40] Sjobakk TE, Vettukattil R, Gulati M, Gulati S, Lundgren S, Gribbestad IS, et al Metabolic profiles of brain metastases. Int J Mol Sci. 2013;14(1):2104–18. doi: 10.3390/ijms14012104 ; PubMed Central PMCID: PMC3565368. 23340650 10.3390/ijms14012104 PMC3565368

[41] Li X, Vigneron DB, Cha S, Graves EE, Crawford F, Chang SM, et al Relationship of MR-derived lactate, mobile lipids, and relative blood volume for gliomas in vivo. AJNR Am J Neuroradiol. 2005;26(4):760–9. . 15814918 PMC7977113

[42] Opstad KS, Murphy MM, Wilkins PR, Bell BA, Griffiths JR, Howe FA. Differentiation of metastases from high-grade gliomas using short echo time 1H spectroscopy. J Magn Reson Imaging. 2004;20(2):187–92. doi: 10.1002/jmri.20093 . 15269942 10.1002/jmri.20093

[43] Zong H, Parada LF, Baker SJ. Cell of origin for malignant gliomas and its implication in therapeutic development. Cold Spring Harb Perspect Biol. 2015;7(5). doi: 10.1101/cshperspect.a020610 . 25635044 10.1101/cshperspect.a020610 PMC4448618

