# Supplementary material for: Exploration of human brain tumour metabolism using pairwise metabolite-metabolite correlation analysis (MMCA) of HR-MAS 1H NMR spectra
Source: PLoS One. 2017 Oct 25;12(10):e0185980. doi: 10.1371/journal.pone.0185980 (PMC5656327; doi:10.1371/journal.pone.0185980)
Supplement: S1 Table — Numbers of samples with information on tissue weights are given in the brackets. (PDF) [file pone.0185980.s001.pdf]

S1 Table. Details of the brain tumour datasets used in this study (number of samples with information of weight are in the brackets)

**Frequency**	**Centre**	**GBM**	**AST**	**MN**	**ODG**	**MET**	TOTAL	MAS spin rate & Sample temperature
**500 MHz**	**UVEG**	17 (16)	4 (4)	9 (9)	3 (3)	6 (6)		4 kHz & 0°C
	**CDP-IAT**	20 (20)	20 (19)	2 (1)	5 (5)	3 (3)		4 kHz & 0°C
	**IDI-BAD**	2 (2)	1(1)	0	1 (1)	2 (2)		4 kHz & 0°C
	**FLENI**	8 (8)	9 (9)	7 (7)	5 (5)	2 (2)		4 kHz & 4°C
	**MUL**	7 (7)	4 (4)	8 (8)	2 (2)	3 (3)		4 kHz & °C
	**IDI-BELL**	21 (21)	9 (9)	18 (18)	1 (1)	1 (1)		4 kHz & 4°C
	**UMCN**	10 (10)	8 (8)	7 (7)	4 (3)	5 (5)		2.5 kHz & 4°C
	**HSJD**		1 91)					unknown
**600 MHz**	**FLENI**	26 (24)	30 (30)	10 (10)	15 (14)	2 (2)		4 kHz & 4°C
	**SGUL**	21(20)	15 (14)	13 (8)	1 (1)	9 (9)		5 kHz & 4°C
	**UMCN**			1 (1)				2.5KHz & 4°C
	Total	132(128)	101(99)	75 (69)	37 (35)	33 (33)	378 (364)	

Metastatic tumours details	
Primary origin	Number of samples
Lung	8
Breast	2
Renal	1
Oesophagus	1
No information	21
Total	33

GBM – glioblastoma, AST - astrocytoma, MN - meningioma, ODG- oligodendroglioma, MET - metastasis

Universidad de Valencia (UVEG), Valencia, Spain ; CDP-IAT Centre Diagnòstic Pedralbes (CDP)- Institut d'Alta Tecnologia (IAT) Barcelona, Spain, IDI-BAD - Institut de Diagnòstic per la Imatge (IDI)-Badalona in Barcelona, Spain ;   Fundacio´n Lucha contra las Enfermedades Neurolo´ gicas de la Infancia (FLENI), Buenos Aires, Argentina; Medical University of Lodz (MUL), Lodz, Poland; Bellvitge Biomedical Research Institute (IDIBELL), Barcelona – Spain; University Medical Centre Nijmegen(UCMN), Nijmegen , The Netherlands; Hospital Sant Joan de De´u (HSJD), Barcelona, Spain; St Georges' Hospital and Medical School (SGUL), London, UK.
