# Supplementary material for: Exploration of human brain tumour metabolism using pairwise metabolite-metabolite correlation analysis (MMCA) of HR-MAS 1H NMR spectra
Source: PLoS One. 2017 Oct 25;12(10):e0185980. doi: 10.1371/journal.pone.0185980 (PMC5656327; doi:10.1371/journal.pone.0185980)
Supplement: S2 Table — Data from http://www.cancer.org/index and www.abta.org/. (PDF) [file pone.0185980.s002.pdf]

Type of Tumour	5-Year Relative Survival Rate		
	Age		
	20-44	45-54	55-64
Low-grade (diffuse) astrocytoma	65%	43%	21%
Anaplastic astrocytoma	49%	29%	10%
Glioblastoma	17%	6%	4%
Oligodendroglioma	85%	79%	64%
Anaplastic oligodendroglioma	67%	55%	38%
Ependymoma/anaplastic ependymoma	91%	86%	85%
Meningioma	92%	77%	67%
