# Supplementary material for: Exploration of human brain tumour metabolism using pairwise metabolite-metabolite correlation analysis (MMCA) of HR-MAS 1H NMR spectra
Source: PLoS One. 2017 Oct 25;12(10):e0185980. doi: 10.1371/journal.pone.0185980 (PMC5656327; doi:10.1371/journal.pone.0185980)
Supplement: S3 Table — Data adapted from ref [7]. (PDF) [file pone.0185980.s003.pdf]

S3 Table. Correlation coefficients of metabolites in control and E1A/RAS treated samples (data adapted from ref [7])

| | | Control | E1A/RAS treated |
|---|---|---|---|
| **Glycolysis** | Lac and Ala | 0.49 | 0.78 |
| **Glutamine and glutamate metabolism** | Gln and Glu | 0.06 | 0.22 |
| | tGlu and Lac | -0.68 | -0.88 |
| | tGlu and tCho | 0.19 | 0.72 |
| **Energy metabolism** | tCr and Lac | 0.54 | -0.48 |
| | tCr and Ala | 0.72 | 0.01 |
| | tCr and tCho | -0.20 | 0.42 |
| **Membrane metabolism** | PCh and GPC | -0.25 | 0.29 |
| | tCho and Lac | -0.34 | -0.83 |
