# Supplementary figures and images for: Exploration of human brain tumour metabolism using pairwise metabolite-metabolite correlation analysis (MMCA) of HR-MAS 1H NMR spectra
Source: PLoS One. 2017 Oct 25;12(10):e0185980. doi: 10.1371/journal.pone.0185980 (PMC5656327; doi:10.1371/journal.pone.0185980)

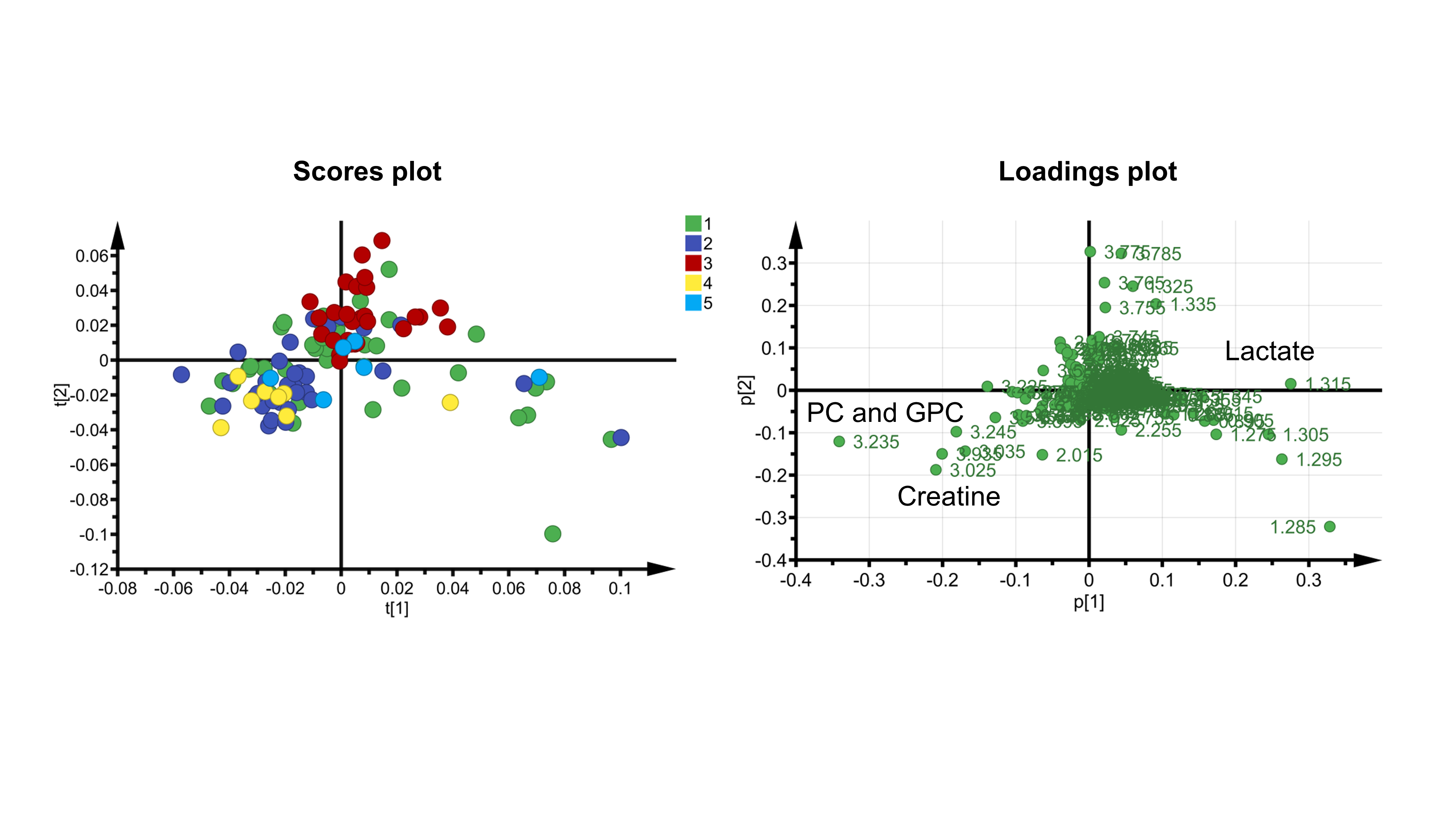

Supplement: S1 Fig — 1. Glioblastoma (green dots, n = 44), 2. Astrocytoma (violet dots, n = 31), 3. Meningioma (red dots, n = 19), 4. Oligodendroglioma (yellow dots, n = 7) and 5. Metastasis (blue dots, n = 5) samples. (TIF) [file pone.0185980.s004.tif]

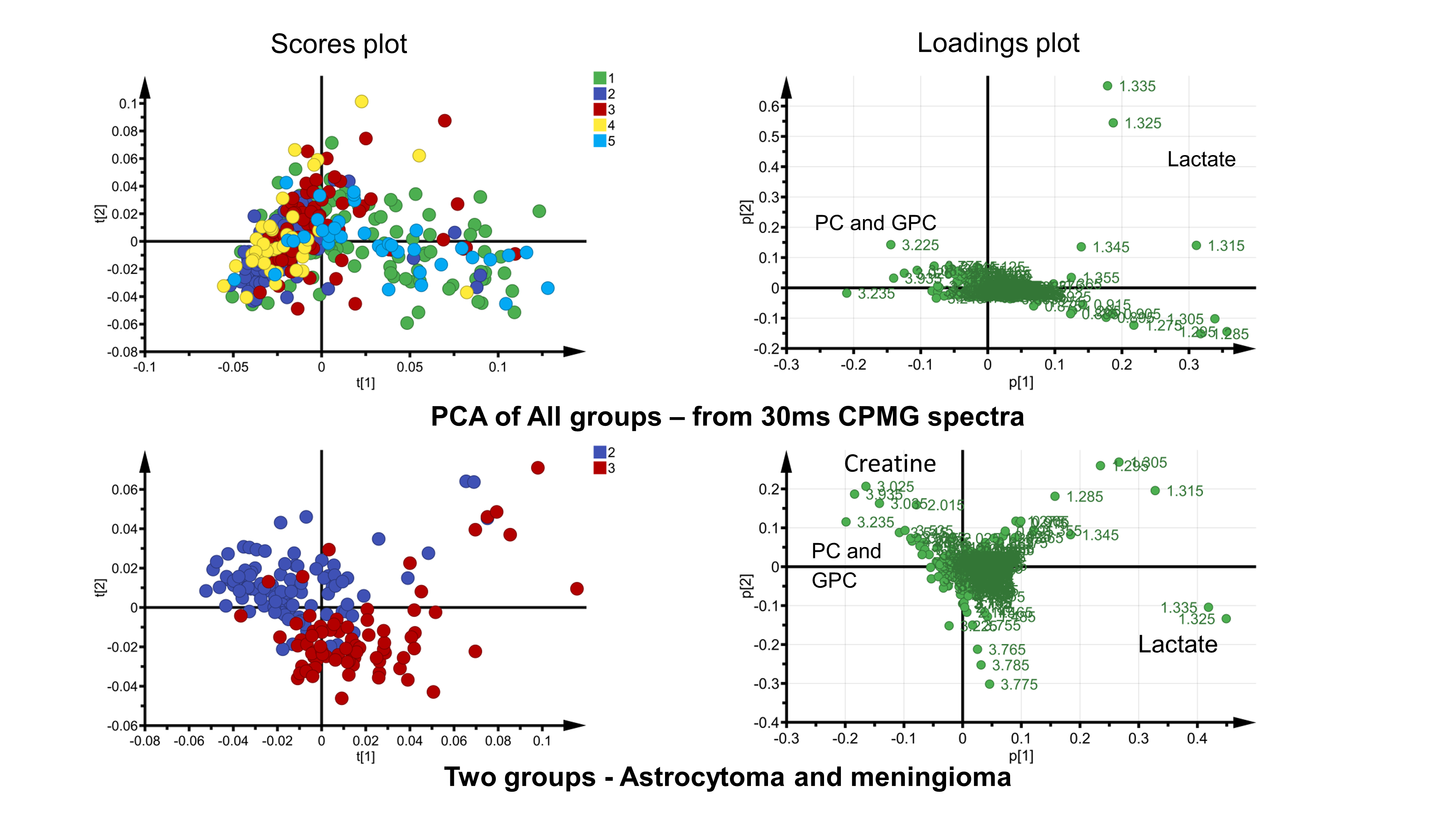

Supplement: S2 Fig — 1. Glioblastomas (green dots, n = 145), 2. Astrocytoma (violet dots, n = 101), 3. Meningioma (red dots, n = 75), 4. Oligodendroglioma (yellow dots, n = 37) and 5. Metastasis (blue dots, n = 33). (TIF) [file pone.0185980.s005.tif]

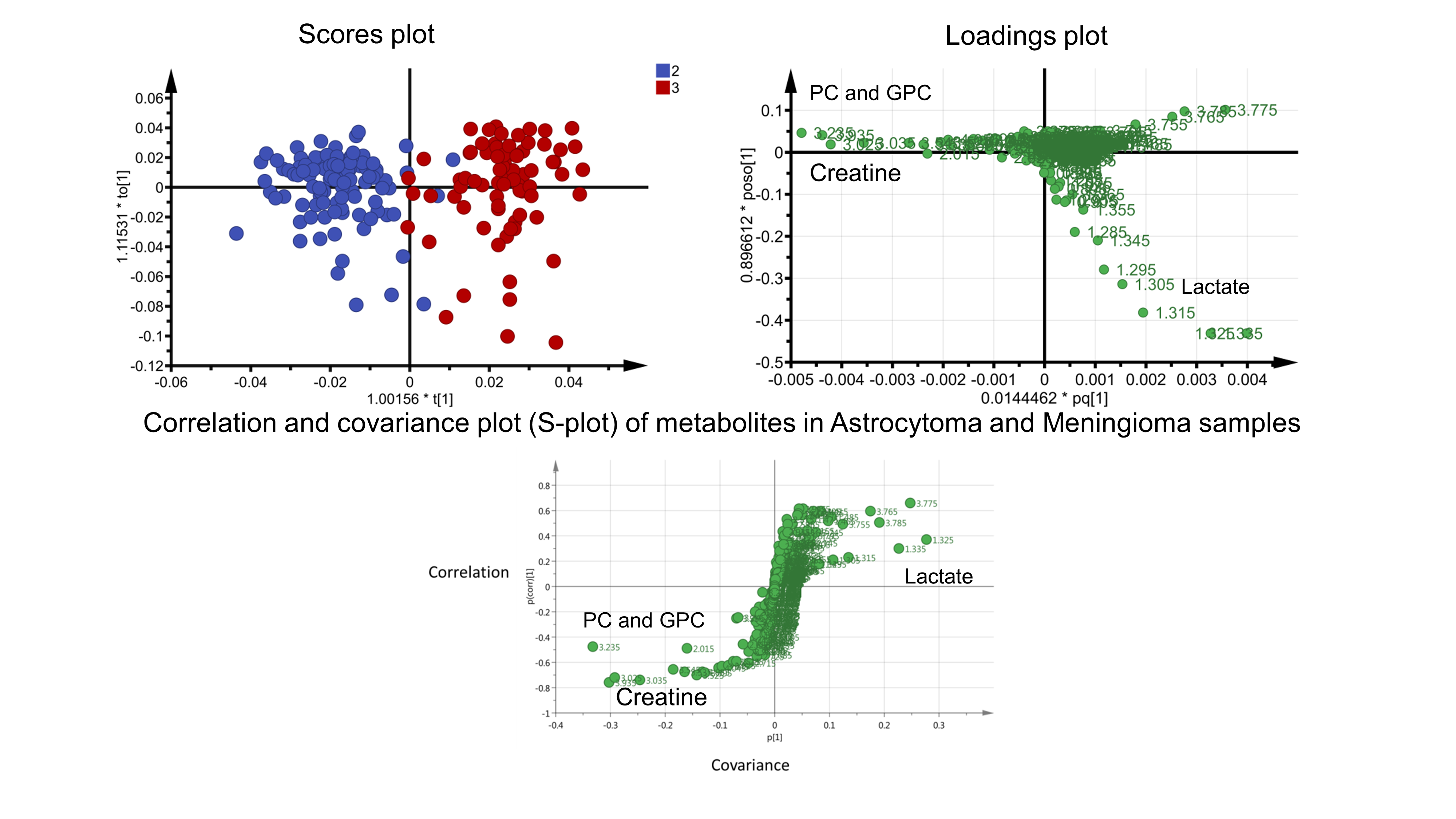

Supplement: S3 Fig — Astrocytoma (violet dots, n = 101), Meningioma (red dots, n = 75) samples. (TIF) [file pone.0185980.s006.tif]

**A**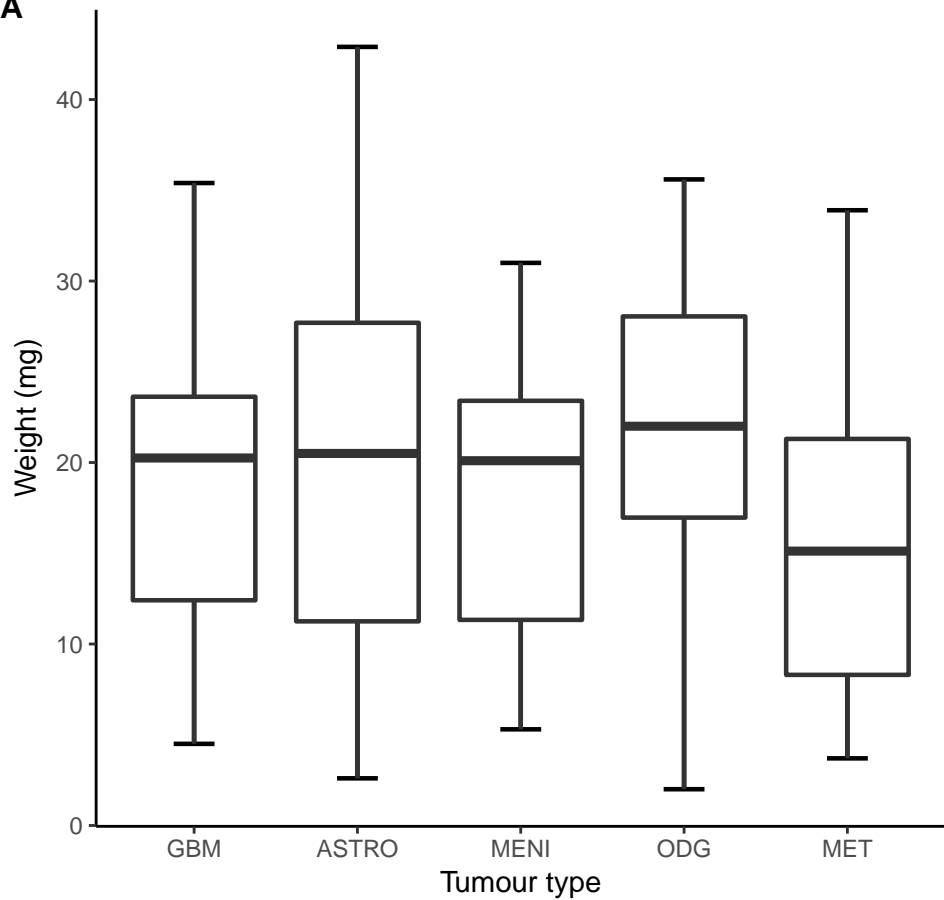

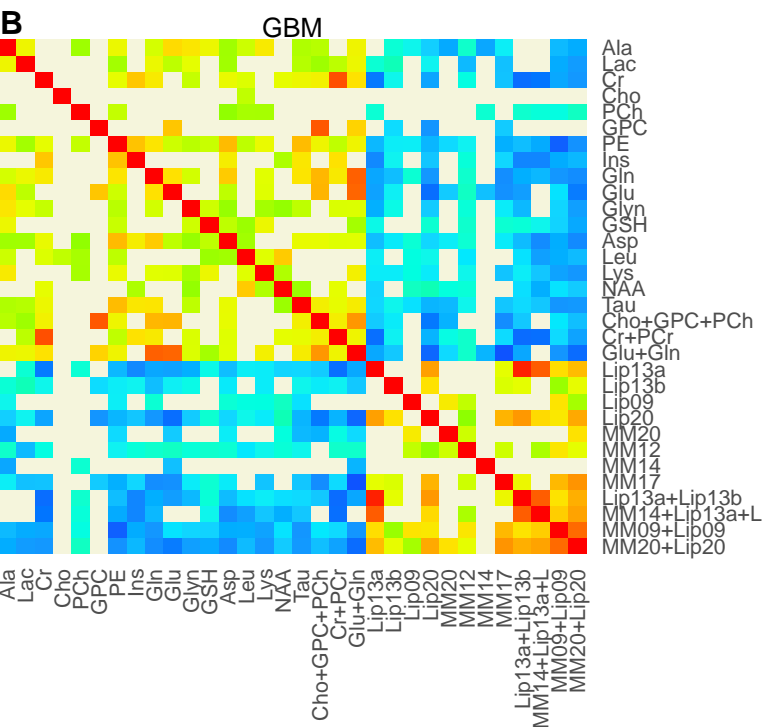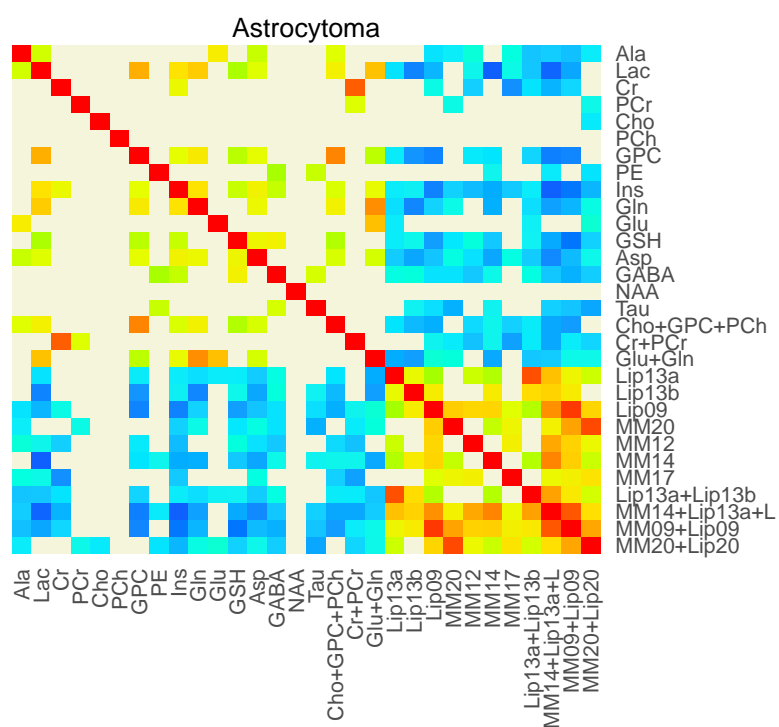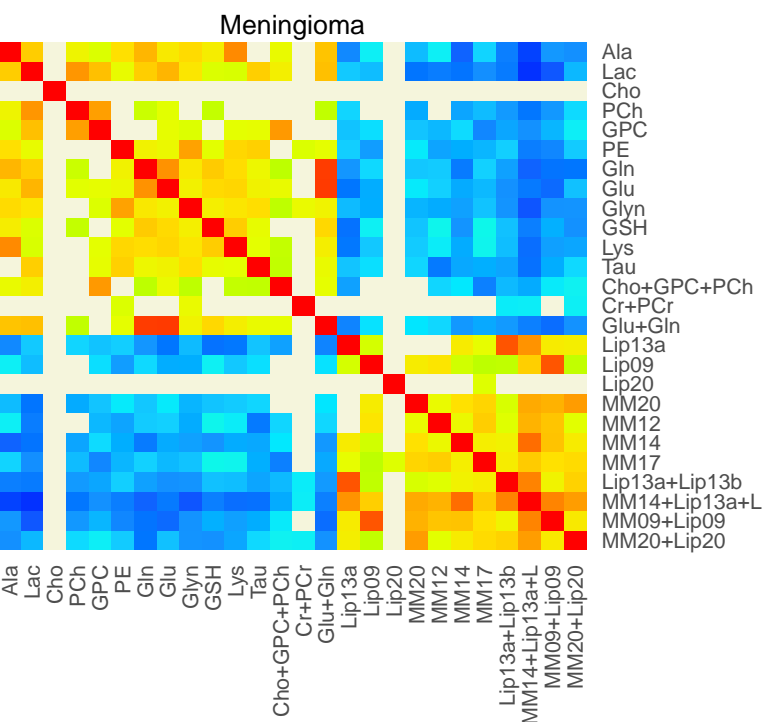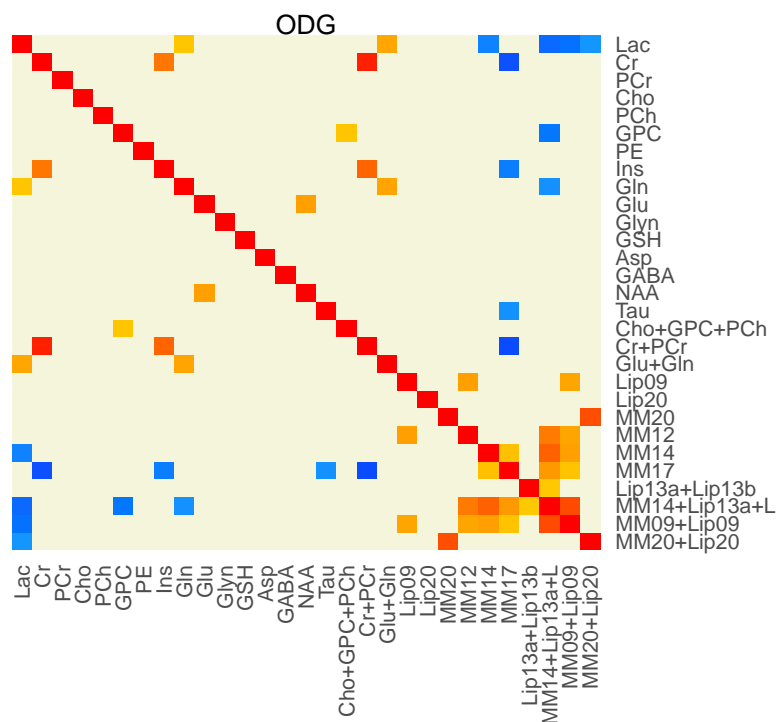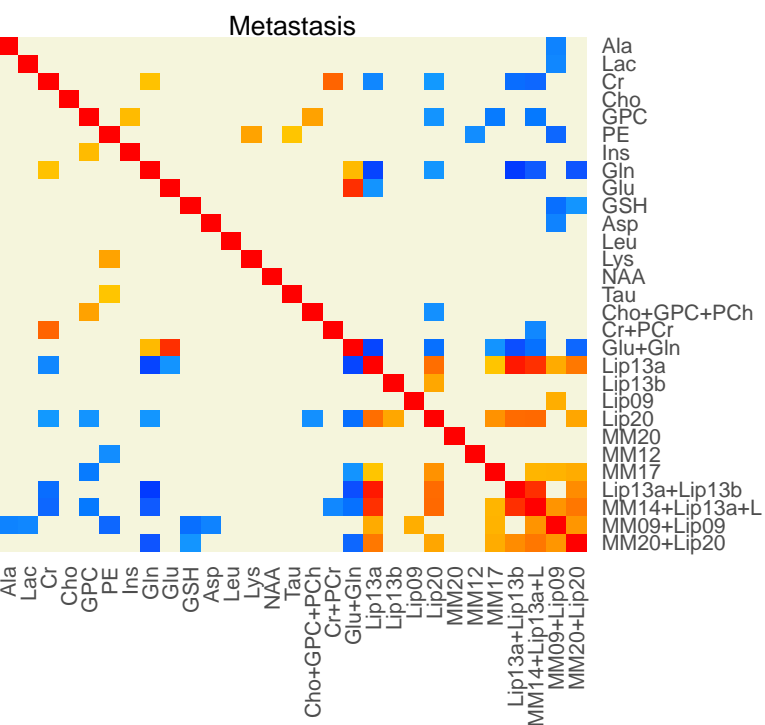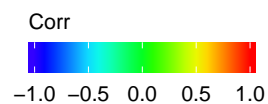

C

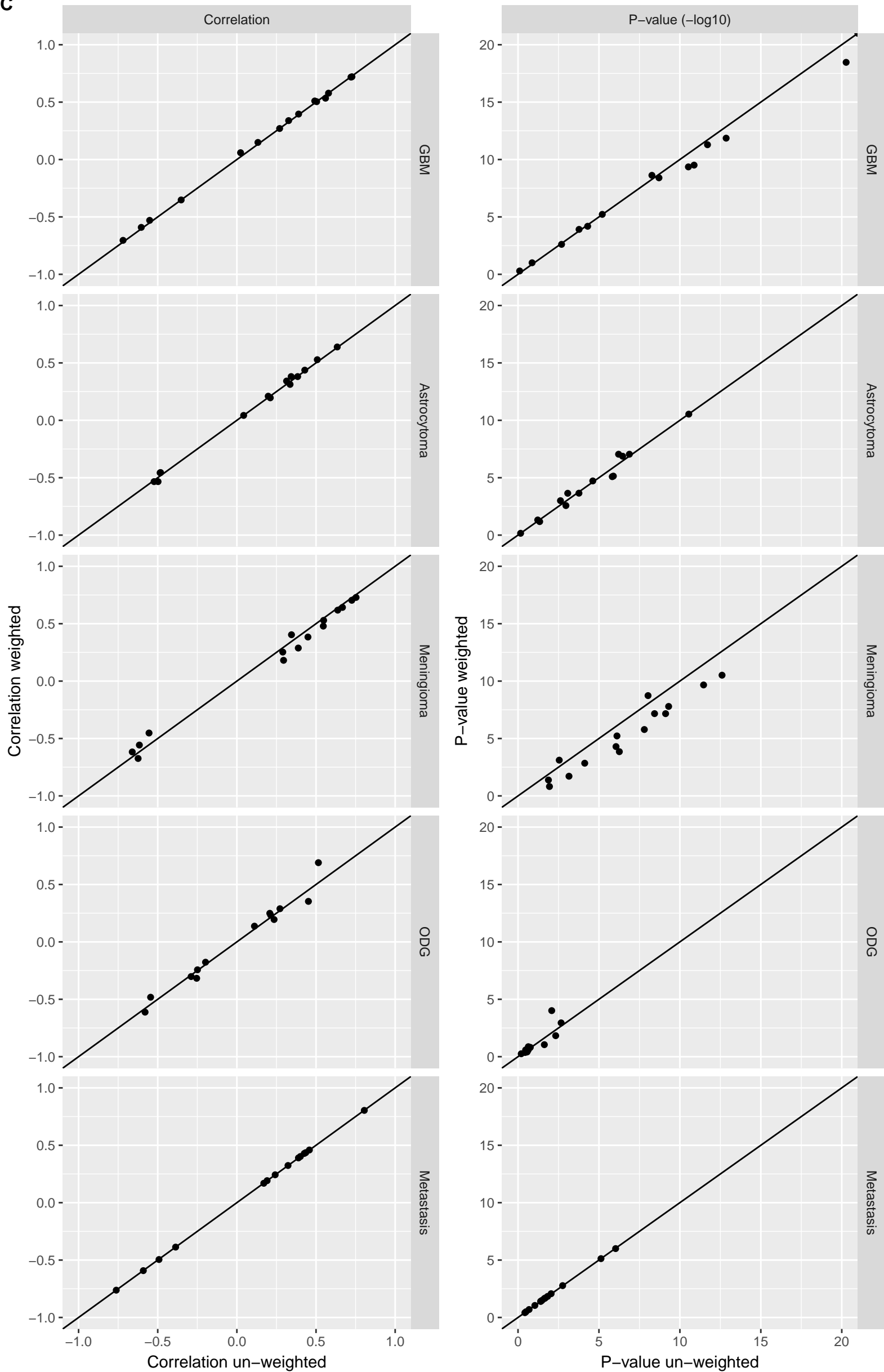

Supplement: S4 Fig — (A) Boxplots illustrating the median and range for tissue weights across the five different tumour types. (B) MMCA heatmaps based on the tissue normalised data from the five tumour types. (C) Comparison of non-weighted and weighted correlation coefficients and p-values across the five tumour types. The heatmaps were filtered using a p-value cut-off of 0.001 coupled to a bootstrap confidence interval of level 0.999. (PDF) [file pone.0185980.s007.pdf]

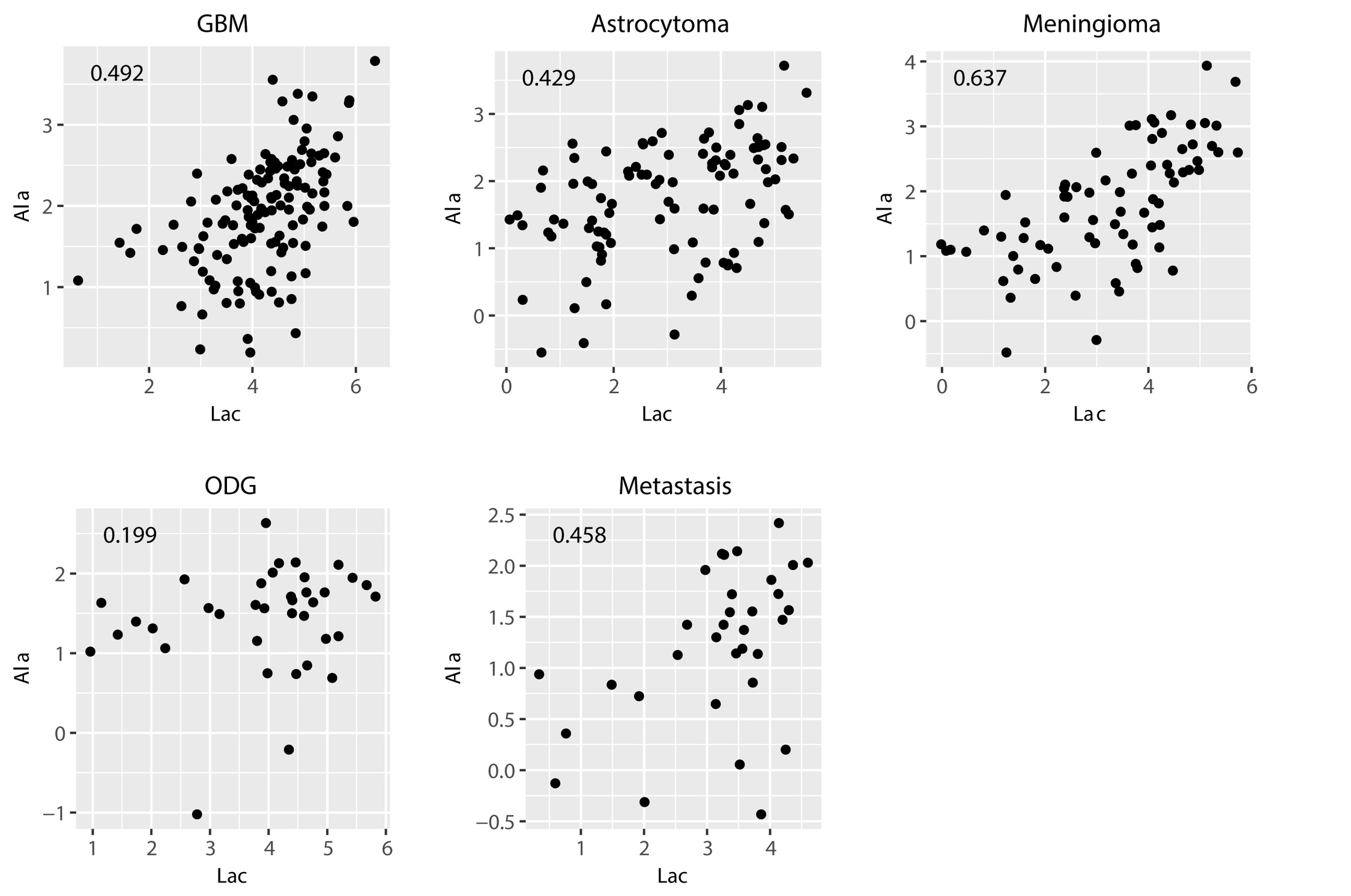

Supplement: S5 Fig — (TIF) [file pone.0185980.s008.tif]

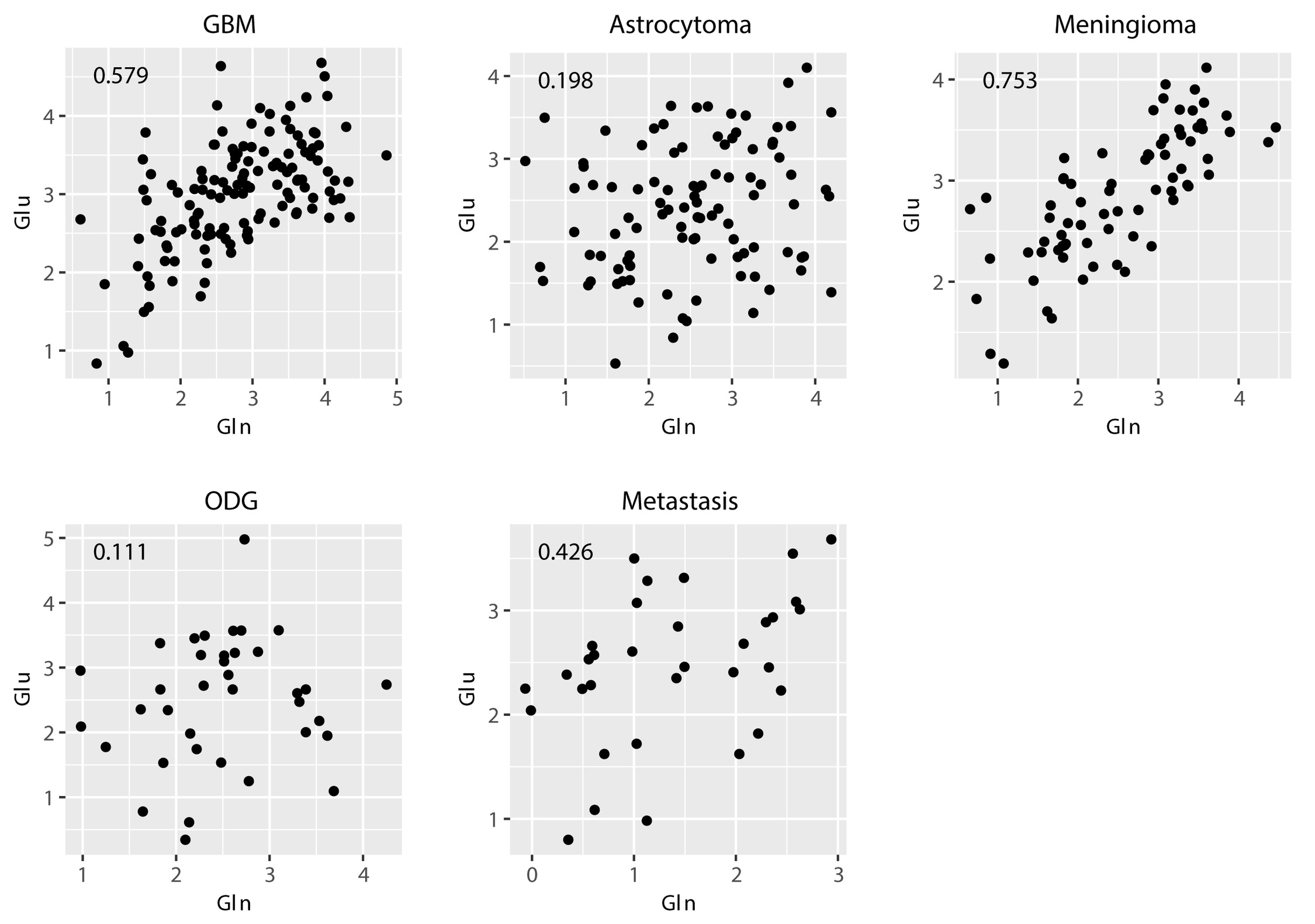

Supplement: S6 Fig — (TIF) [file pone.0185980.s009.tif]

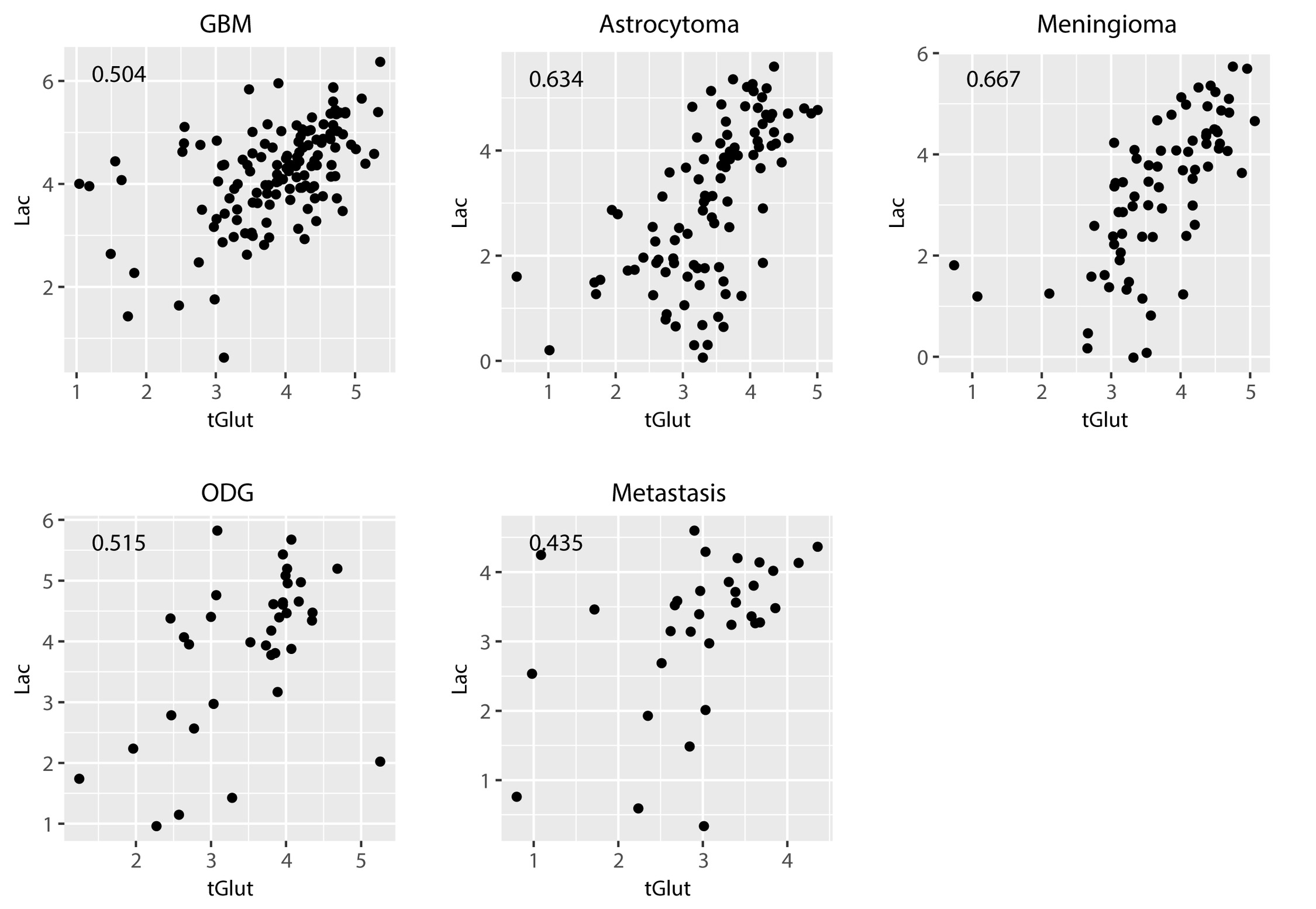

Supplement: S7 Fig — (TIF) [file pone.0185980.s010.tif]

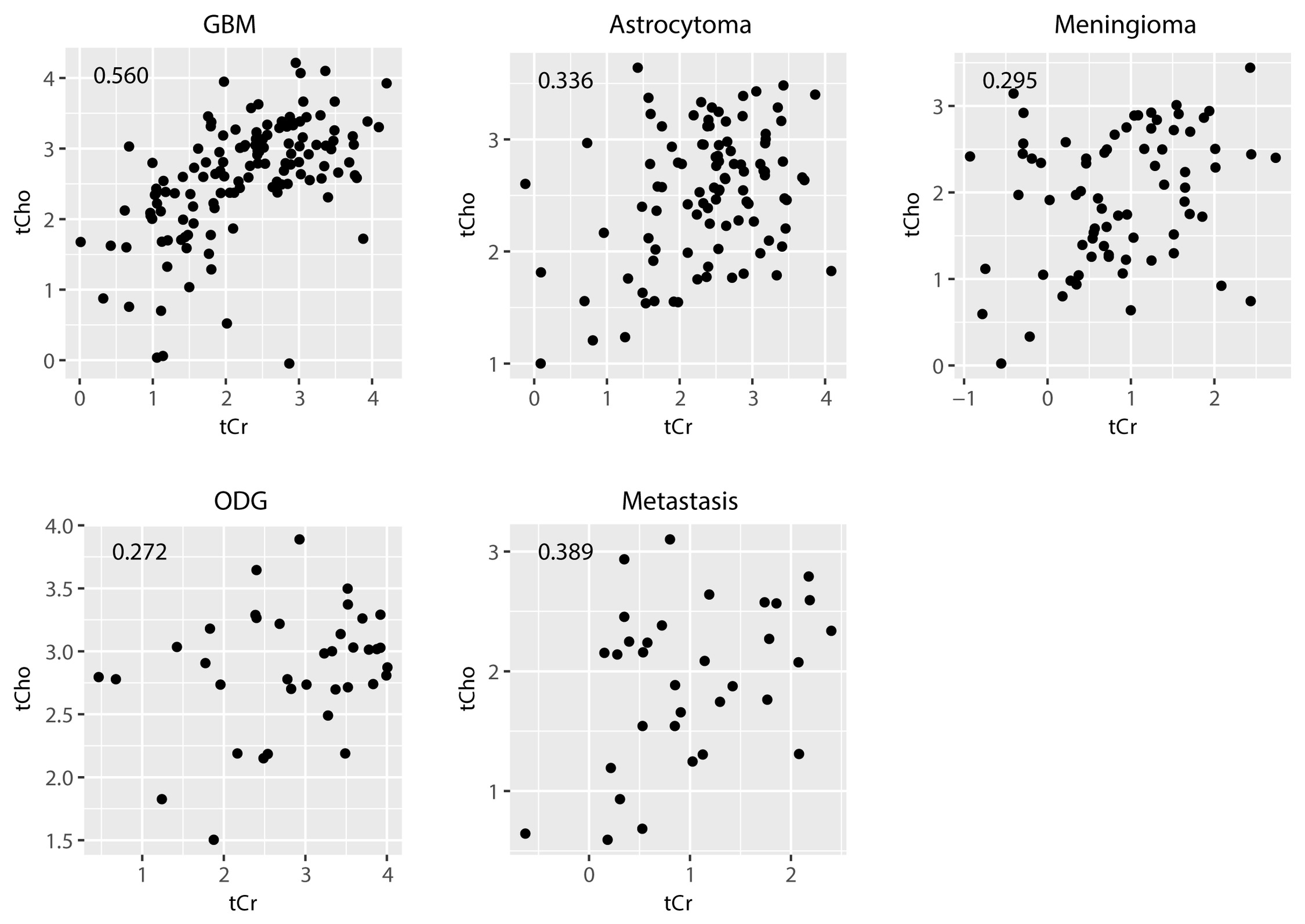

Supplement: S8 Fig — (TIF) [file pone.0185980.s011.tif]

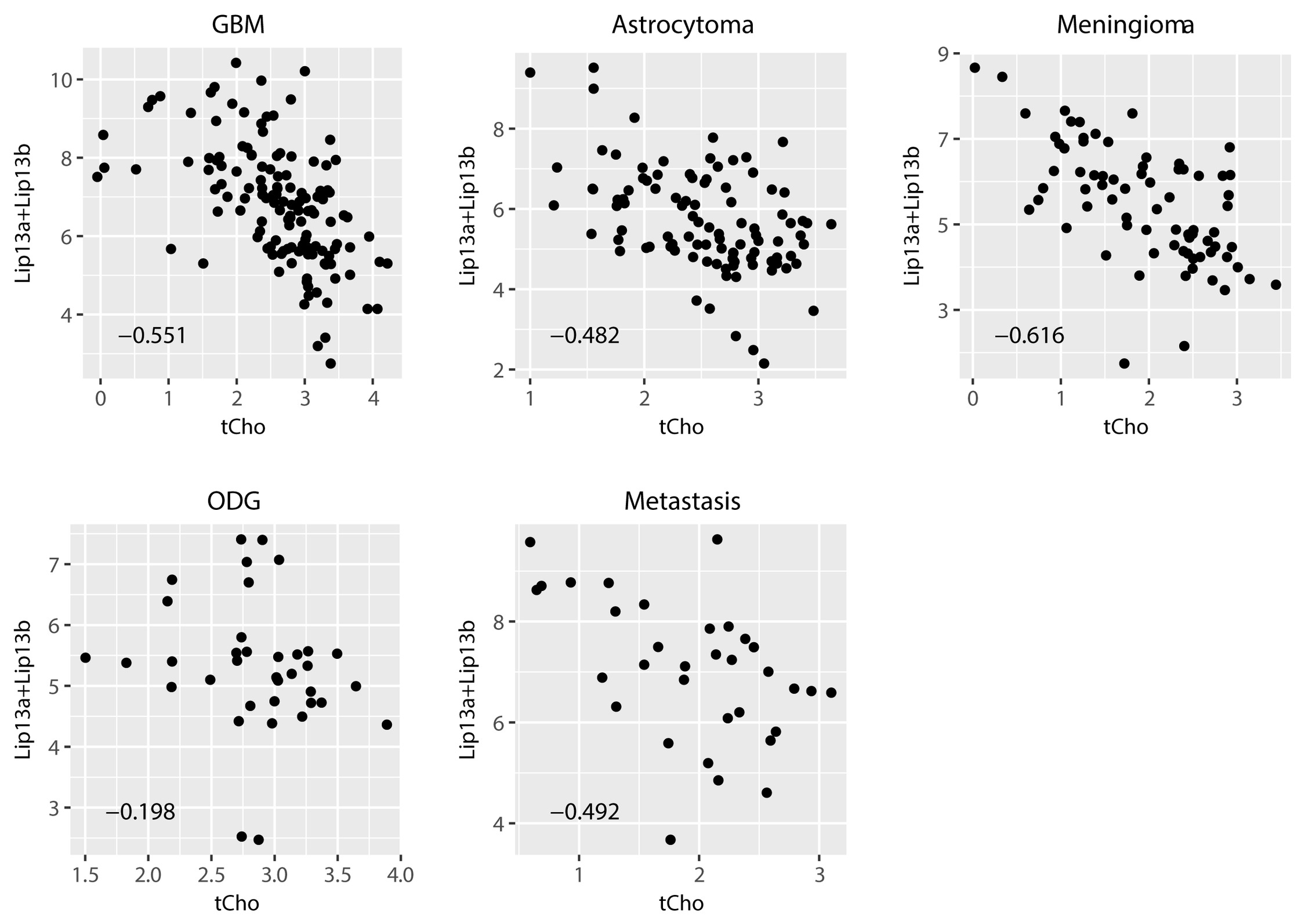

Supplement: S9 Fig — (TIF) [file pone.0185980.s012.tif]

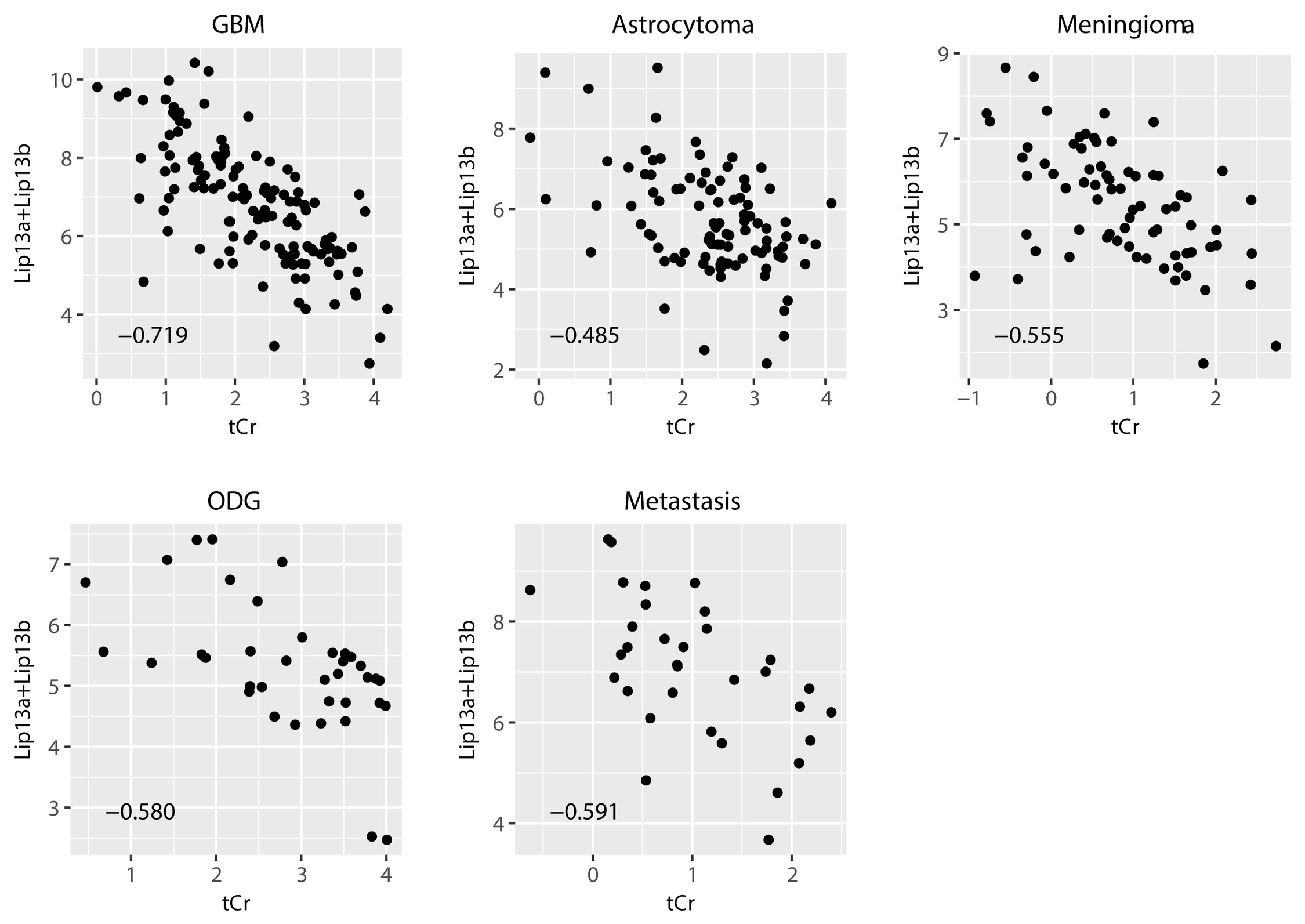

Supplement: S10 Fig — (TIF) [file pone.0185980.s013.tif]

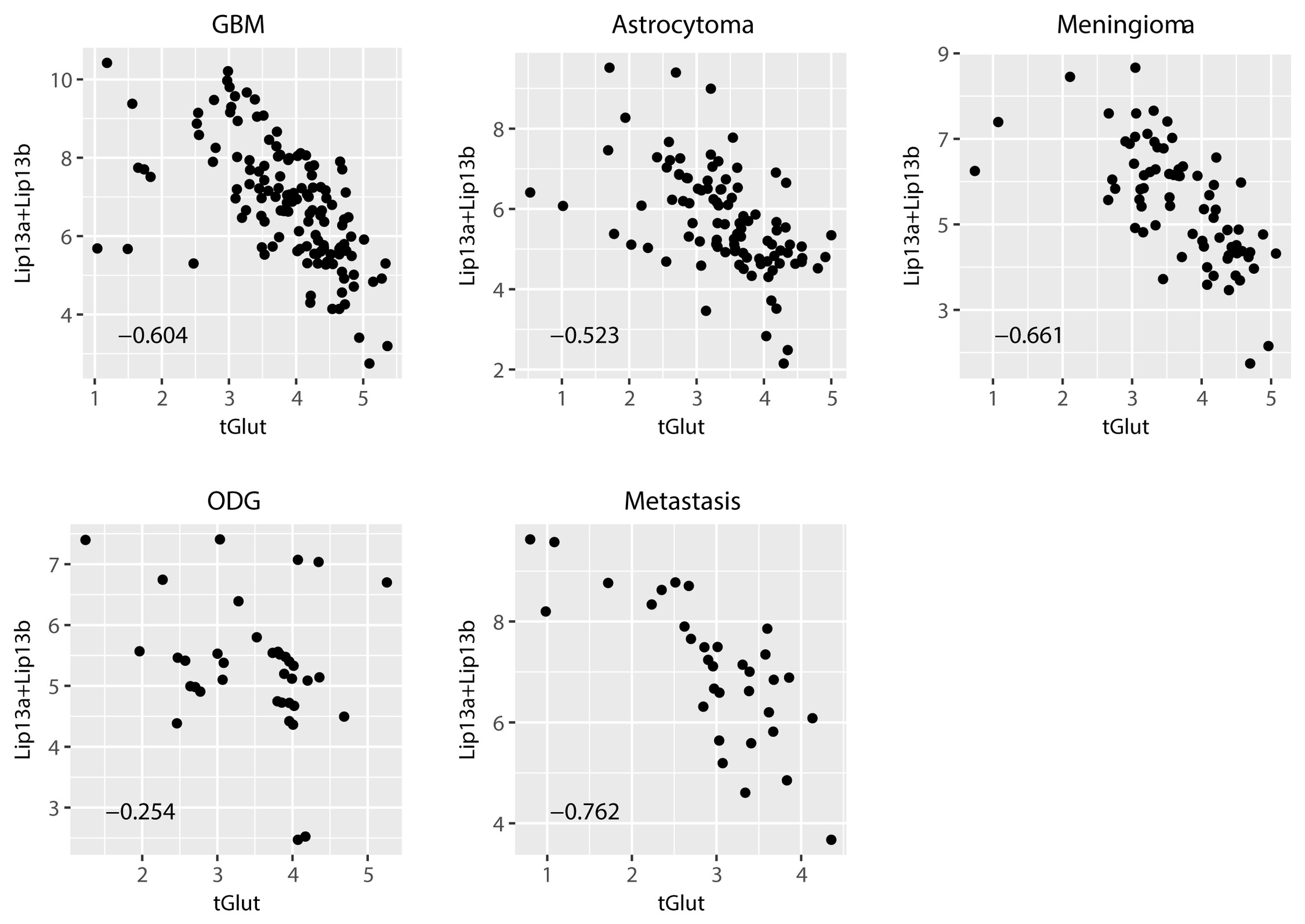

Supplement: S11 Fig — (TIF) [file pone.0185980.s014.tif]
